# Association of school absence and exclusion with recorded neurodevelopmental disorders, mental disorders, or self-harm: a nationwide, retrospective, electronic cohort study of children and young people in Wales, UK

**DOI:** 10.1016/S2215-0366(21)00367-9

**Published:** 2022-01

**Authors:** Ann John, Yasmin Friedmann, Marcos DelPozo-Banos, Aura Frizzati, Tamsin Ford, Anita Thapar

**Affiliations:** a Swansea University Medical School, Swansea University, Swansea, UK; b Cedar Healthcare Technology Research Centre, Cardiff Medicentre, University Hospital of Wales, Cardiff, UK; c Department of Psychiatry, University of Cambridge, Cambridge, UK; d Division of Psychological Medicine and Clinical Neurosciences, Cardiff University, Cardiff, UK

## Abstract

**Background:**

Poor attendance at school, whether due to absenteeism or exclusion, leads to multiple social, educational, and lifelong socioeconomic disadvantages. We aimed to measure the association between a broad range of diagnosed neurodevelopmental and mental disorders and recorded self-harm by the age of 24 years and school attendance and exclusion.

**Methods:**

In this nationwide, retrospective, electronic cohort study, we drew a cohort from the Welsh Demographic Service Dataset, which included individuals aged 7–16 years (16 years being the school leaving age in the UK) enrolled in state-funded schools in Wales in the academic years 2012/13–2015/16 (between Sept 1, 2012, and Aug 31, 2016). Using the Adolescent Mental Health Data Platform, we linked attendance and exclusion data to national demographic and primary and secondary health-care datasets. We identified all pupils with a recorded diagnosis of neurodevelopmental disorders (ADHD and autism spectrum disorder [ASD]), learning difficulties, conduct disorder, depression, anxiety, eating disorders, alcohol or drugs misuse, bipolar disorder, schizophrenia, other psychotic disorders, or recorded self-harm (our explanatory variables) before the age of 24 years. Outcomes were school absence and exclusion. Generalised estimating equations with exchangeable correlation structures using binomial distribution with the logit link function were used to calculate odds ratios (OR) for absenteeism and exclusion, adjusting for sex, age, and deprivation.

**Findings:**

School attendance, school exclusion, and health-care data were available for 414 637 pupils (201 789 [48·7%] girls and 212 848 [51·3%] boys; mean age 10·5 years [SD 3·8] on Sept 1, 2012; ethnicity data were not available). Individuals with a record of a neurodevelopmental disorder, mental disorder, or self-harm were more likely to be absent or excluded in any school year than were those without a record. Unadjusted ORs for absences ranged from 2·1 (95% CI 2·0–2·2) for those with neurodevelopmental disorders to 6·6 (4·9–8·3) for those with bipolar disorder. Adjusted ORs (aORs) for absences ranged from 2·0 (1·9–2·1) for those with neurodevelopmental disorders to 5·5 (4·2–7·2) for those with bipolar disorder. Unadjusted ORs for exclusion ranged from 1·7 (1·3–2·2) for those with eating disorders to 22·7 (20·8–24·7) for those with a record of drugs misuse. aORs for exclusion ranged from 1·8 (1·5–2·0) for those with learning difficulties to 11·0 (10·0–12·1) for those with a record of drugs misuse.

**Interpretation:**

Children and young people up to the age of 24 years with a record of a neurodevelopmental or mental disorder or self-harm before the age of 24 years were more likely to miss school than those without a record. Exclusion or persistent absence are potential indicators of current or future poor mental health that are routinely collected and could be used to target assessment and early intervention. Integrated school-based and health-care strategies to support young peoples' engagement with school life are required.

**Funding:**

The Medical Research Council, MQ Mental Health Research, and the Economic and Social Research Council.

**Translation:**

For the Welsh translation of the abstract see Supplementary Materials section.

## Introduction

Poor school attendance due to absence (authorised or unauthorised) from available sessions or exclusion (where a headteacher forbids a student to attend for a fixed number of sessions or permanently) leads to multiple immediate and long-term socioeconomic disadvantages. It is associated with a range of negative outcomes across the life course, including poor educational attainment, unemployment, and poverty.^1^, ^2^, ^3^, ^4^, ^5^ Several small-scale studies in the UK, USA, and Australia, with sample sizes ranging from less than 100 to 13 000, suggest that absence from school is more common in children with a mental disorder, specifically depression, anxiety, and disruptive behaviour disorders, through school refusal, truancy, or the condition itself.^6^, ^7^, ^8^, ^9^, ^10^, ^11^ Studies from the UK^12^, ^13^ and the USA^14^ report an association between neurodevelopmental disorders (ie, ADHD and autism spectrum disorder [ASD]) and self-harm with persistent absenteeism. Similarly, school exclusion appears to be strongly associated with ADHD, ASD, and mental disorders, particularly depression, in UK-based and international studies.^13^, ^15^ In these mostly cross-sectional studies, diagnoses were assessed by use of questionnaires or interviews. However, children and young people (≤24 years) with these disorders are more commonly from disadvantaged families and might be less likely to participate in research surveys.^16^, ^17^, ^18^ They also have higher levels of attrition at follow-up^16^, ^17^, ^18^ for reasons including impairments affecting the young person or their parent and impacting survey completion or a related absence when surveys are done in a school setting. Furthermore, birth cohort studies often include insufficient numbers of children with mental health conditions to support in-depth analysis of rarer conditions.

**Research in context**

**Evidence before this study**

We searched PubMed for papers published in English between database inception and July, 20, 2021, using the search terms ((children) OR (adolescents)) AND ((school attendance) OR (school absence) OR (exclu*) OR (truan*) or (school disengagement) OR (School Refusal)) AND ((depression) or (anxiety) or (adhd) or (autism) or (learning difficulty) or (schizophrenia) or (bipolar) or (self-harm) or (eating disorder) or (drugs) or (alcohol) or (conduct disorder)). We found 13 small-scale cross-sectional surveys that used questionnaires to assess mental disorders and one national electronic cohort study linking education and secondary health-care datasets. School absence and exclusion were found to be associated with neurodevelopmental disorders, depression, anxiety, disruptive behaviour, substance misuse, or self-harm, but current evidence is sparse and based on small numbers.

**Added value of this study**

Our population-based, electronic cohort study was larger than most previous studies, including more than 400 000 pupils, and linked routinely collected primary and secondary health-care data to educational data. Previous studies have been based on secondary care data only and probably missed disorders, such as anxiety, that are more commonly managed in primary care. Our study encompasses a wide range of clinically diagnosed and recorded mental and neurodevelopmental disorders up to the age of 24 years, and so includes conditions, such as bipolar disorder and schizophrenia, that are less frequently studied in this context are are more often diagnosed in late adolescence and early adulthood. Furthermore, the large size of this study allows for the inclusion of people with less common diagnoses, such as eating disorders. We found strong associations across all disorders and self-harm with absenteeism and exclusion from school. Odds ratios for both outcomes increased with the number of comorbidities and deprivation.

**Implications of all the available evidence**

Poor attendance affects the educational attainment of children and future social and developmental outcomes. Children with mental or neurodevelopmental disorders or who self-harm are more likely to miss school through absenteeism and exclusion than their peers. Exclusion or persistent absence are potential indicators for current or future poor mental health that are routinely collected and could be used to target assessment and early intervention.

In this study, we capitalised on electronic linkage between routinely collected primary and secondary health-care data on clinical diagnoses and data on school attendance and exclusions at a population level. Our hypothesis was that school absences and exclusions are associated with a broad range of diagnosed and recorded neurodevelopmental and mental disorders and self-harm by 24 years of age within our cohort of pupils, even after adjusting for sex, age at the start of the academic year, and deprivation. Once established (and previous literature is scarce), this hypothesis would lead to further questions for more detailed study.

## Methods

### Study design and participants

In this nationwide, retrospective, electronic cohort study, we drew our cohort from the 5 341 392 individuals in the Welsh Demographic Service Dataset to include individuals aged 7–16 years (16 years being the school leaving age in the UK) enrolled in state-funded schools in Wales in the academic years 2012/13–2015/16 (between Sept 1, 2012, and Aug 31, 2016) who had primary and secondary care linked data and no conflicting data in the education dataset that pointed to a many-to-one correspondence between the anonymised linkage field and the internal pupil identification number. Ethics approval was granted from the Secure Anonymised Information Linkage (SAIL) Information Governance Review Panel, an independent body consisting of a range of government, regulatory, and professional agencies, in line with ethical permissions already granted to the analysis of data in the SAIL Databank (approval number 0808).

### Procedures

We linked data on an individual level via the Adolescent Mental Health Data Platform, an international data platform that supports mental health research in children and young people. For our study, the Adolescent Mental Health Data Platform used datasets from the SAIL Databank, a repository of routinely collected health and education datasets for the population of Wales.^19^, ^20^ All data are treated in accordance with the Data Protection Act 2018. Individuals within the datasets are assigned a unique anonymised linkage field that replaces any identifiable information, such as names, and enables anonymised linkage across the different datasets.

The datasets in the SAIL Databank that we used were: the Welsh Demographic Service Dataset (a demographics register of people registered with general practitioner [GP] practices in Wales) on Nov 1, 2018; the Office for National Statistics deaths register on March 28, 2019; the Welsh Index of Multiple Deprivation 2011 (an official measure of small area [defined as containing approximately 1500 individuals] deprivation in Wales, based on employment opportunities, income, education, health, community safety, geographical access to services, housing, and the physical environment; quintile 5 represents the most deprived areas) on Nov 1, 2018; the Welsh Longitudinal General Practice Database (on Aug 20, 2018) and the Patient Episode Database for Wales (on Jan 31, 2019), which contain attendance and clinical information for all GP interactions and hospital inpatient and day case activity in Wales, respectively; and the Welsh Government Education Dataset (appendix 2 p 6). The Welsh Government Education Dataset includes records for all children registered at mainstream state schools in Wales or educated in settings other than school. It contains information on attendance, exclusions, eligibility for free school meals, and receipt of a statement of special educational needs (SEN). Attendance records were available from the academic year 2007–08 to the academic year 2015–16. Each school reported, per pupil, the number of authorised and unauthorised absences for that year out of a total number of possible sessions per year. Exclusion records (categorised as permanent, fixed, or lunchtime) were available from the academic year 2012–13 to the academic year 2015–16. A child might have SEN status if they have a learning difficulty or disability (including neurodevelopmental or mental disorders) that requires special education provisions to be made for them.^21^

We queried primary and secondary care datasets to extract recorded neurodevelopmental and mental disorders and self-harm using code lists from the ICD (version 10) for secondary care and read codes (version 2)^22^ in primary care. The codes were collated from published articles and code lists or were compiled in collaboration with clinicians (appendix 2 p 7). Neurodevelopmental disorders (ie, ASD and ADHD), learning difficulties, and conduct disorder were extracted for our cohort of pupils from their birth until they reached 24 years of age because these conditions often arise early in development and are diagnosed at a young age. Other mental disorders (including depression, anxiety, eating disorders, bipolar disorder, schizophrenia, alcohol misuse, and drugs misuse) or self-harm were extracted for our cohort of pupils between the ages of 10 years and 24 years. We categorised all F ICD-10 codes and E read codes not included in other category code lists, such as those for mania, into the other psychotic disorders category. Each pupil had a flag per each disorder categorised as a binary variable (recorded present or absent). The age at first diagnosis was extracted for each pupil and disorder. Where a pupil could not be linked to primary or secondary care datasets, this was flagged as linked or unlinked.

We extracted SEN status for each pupil as a binary variable (present *vs* absent) to understand the extent to which it, in addition to a disorder, affected outcomes. We counted the number of morbidities per person to assess the effect of comorbidities (defined as two or more of the studied disorders recorded for the same individual, not necessarily concurrently).

### Outcomes

We defined absenteeism as a binary variable, categorised as 1 when a pupil missed more than 10% of sessions in 1 year and categorised as 0 otherwise. The choice of 10% was based on a report from Estyn (the quality inspectorate of education in Wales), which described that, of pupils who were absent for more than 10% of sessions, fewer than 80% achieved the level expected of them by age 11 years in mathematics, science, and either English or Welsh as a first language and fewer than 40% achieved level 2 (equivalent to five GCSEs at grades A*–C) at 16 years of age.^23^, ^24^ 10% is the level used in England to define persistent absenteeism,^25^ although the level used in Wales is 20%.^26^ Exclusion (a record of any type of exclusion in a specific academic year) was also categorised as a binary variable (yes *vs* no).

### Statistical analysis

Data were retrieved from the SAIL Databank by use of IBM DB2 9.7 SQL. Statistical analysis was done by use of R (version 3.3.3), accessed through RStudio (version 1.2). We analysed the association between the outcome variables (absenteeism and exclusion) and the existence of a record of each neurodevelopmental or mental disorder and self-harm, up to 24 years of age, using generalised estimating equations (R library geepack).^27^ Generalised estimating equations with exchangeable correlation structures using binomial distribution with the logit link function were used to calculate odds ratios (OR) for absenteeism and exclusion, adjusting for sex, age at the beginning of the academic year, and deprivation. We used a long data format with one row per pupil and year. The experimental unit (id) was the pupil, and the repeated measurements were ordered by academic year (wave). 95% CIs for proportions and percentages were estimated by the Wilson score method with continuity correction. We did not explore causality; therefore, the time dependence of measurements per person (up to four measurements at different academic years) was modelled with a correlation function as a source of variance, which was marginalised over so that the variance of the estimated covariates were calculated efficiently.^28^ We used an exchangeable correlation structure, in which any two measurements for the same pupil had the same correlation. We analysed each recorded disorder separately using a sub-cohort consisting of those presenting with these disorders together with pupils in our cohort with no record of any of these disorders (our controls). We tested multiple models, sequentially adding age (week of birth), sex (male *vs* female), and deprivation quintile (quintile 5 representing the most deprived) as covariates.^29^, ^30^ We tested the goodness of fit for each of these models by calculating the Quasi-likelihood model information criteria.^31^ We stratified the population by condition and analysed the association between the outcome variables and sex, age, and deprivation separately. For the main analysis, we pooled pupils with ADHD and ASD (under neurodevelopmental disorder) and pooled pupils with depression, anxiety, eating disorders, schizophrenia, bipolar disorder, and other psychotic disorders (under any mental disorder).

In the first sensitivity analysis, we ran the main analysis on both the subgroup that had linked health-care data and the full dataset. Neurodevelopmental and mental disorders typically show comorbidity over the life course. We assessed the sensitivity of our analysis to comorbidities by conducting three extra sensitivity analyses. First, we compared the model results between those with one morbidity and those without any of the morbidities studied. Second, we compared the model results between those with more than one morbidity and those without any of the morbidities studied. Finally, separately, we ran the model with the number of comorbidities as a covariate.

We conducted several other sensitivity analyses. For some children, certain types of mental disorders are an entry point for SEN status within the education system. We assessed sensitivity to SEN status by exploring the interaction between any of the disorders studied and SEN status. We also ran our models in the subpopulation of participants first diagnosed or with their first record before 17 years of age (ie, while of school age). Furthermore, we compared individual-level yearly rates of absences (number of sessions absent/total number of possible sessions per year) for those excluded versus for those not excluded and calculated the Pearson correlation coefficient between individual-level yearly rates of absences and individual-level yearly rates of exclusions.

### Role of the funding source

The funders of the study had no role in study design, data collection, data analysis, data interpretation, or writing of the report.

## Results

**Figure 1:**
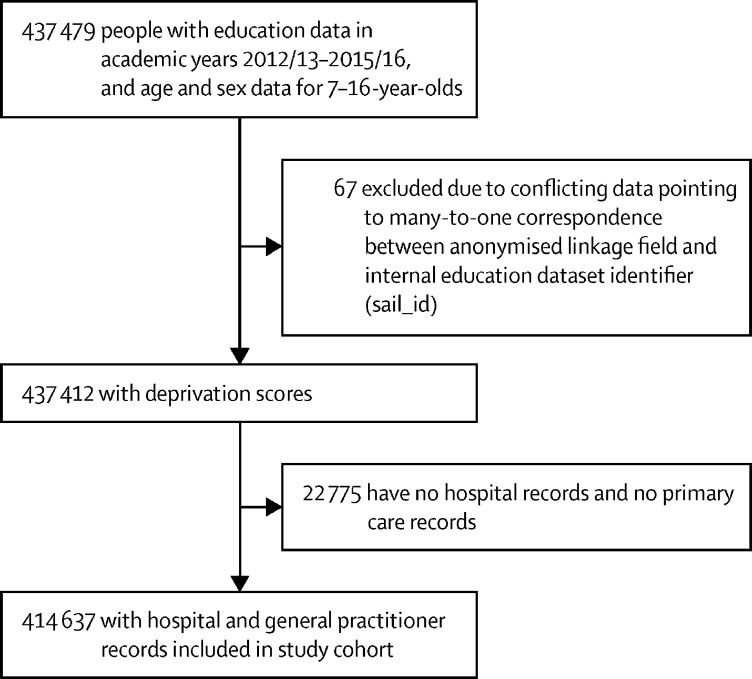
Flow diagram of cohort selection

437 412 individuals had education and demographic data and were aged 7–16 years during the 2013–16 academic years, of whom 213 816 (48·9%) were female and 223 596 (51·1%) were male (figure 1). Of these 437 412 individuals, 22 775 (5·2%) had no linked hospital or primary care records. We considered health data for these individuals as missing at random because missingness was not based on health or education status.

In the group with health-care data, 212 848 (51·3%) of 414 637 pupils were boys and 201 789 (48·7%) were girls (appendix 2 p 1). In the group without health-care data, 10 748 (47·2%) of 22 775 pupils were boys and 12 027 (52·8%) were girls (appendix 2 p 1). Compared with those with health-care data, a higher proportion of individuals with missing health-care data resided in quintile 2 areas on the Welsh Index of Multiple Deprivation and a lower proportion resided in quintile 5 areas (appendix 2 p 1). We repeated the main analysis in the group with linked primary and secondary health-care data (n=414 637) and in the larger group (n=437 412). Results were equivalent (appendix 2 pp 25–26) so we removed those without linked health-care data from all main and other sensitivity analyses (list-wise deletion). Each pupil contributed 1–4 years of data. The distribution of morbidity, sex, and deprivation by number of years of data contributed is shown in appendix 2 (pp 2–4). Demographics and morbidity were not correlated with the number of years of data contributed so we also viewed this as missing at random.

Of the 414 637 pupils with primary and secondary care data comprising our study population, 201 789 (48·7%) were female and 212 848 (51·3%) were male. Their mean age on Sept 1, 2012, was 10·5 years (SD 3·8). Ethnicity data were not available. 57 930 (14·0%) pupils had at least one of the disorders studied (a neurodevelopmental or mental disorder or a record of self-harm) by the age of 24 years, and 42 734 (10·3%) while of school age. 356 707 (86·0%) of 414 637 individuals had no record of self-harm or any of the disorders studied. The numbers of diagnosed individuals, age at first diagnosis, and SEN status before 17 years of age are detailed in appendix 2 (p 7).

118 140 (28·5%) had recorded absenteeism during at least 1 school year, of whom 5901 (5·0%) had a neurodevelopmental disorder, 17 724 (15·0%) had a mental disorder, and 5164 (4·4%) had a record of self-harm. 20 507 (17·4%) of the 118 140 with recorded absenteeism were diagnosed while of school age, of whom 5762 (28·1%) had a neurodevelopmental disorder, 12 164 (59·3%) had a mental disorder, and 4450 (21·7%) had a record of self-harm.

**Figure 2:**
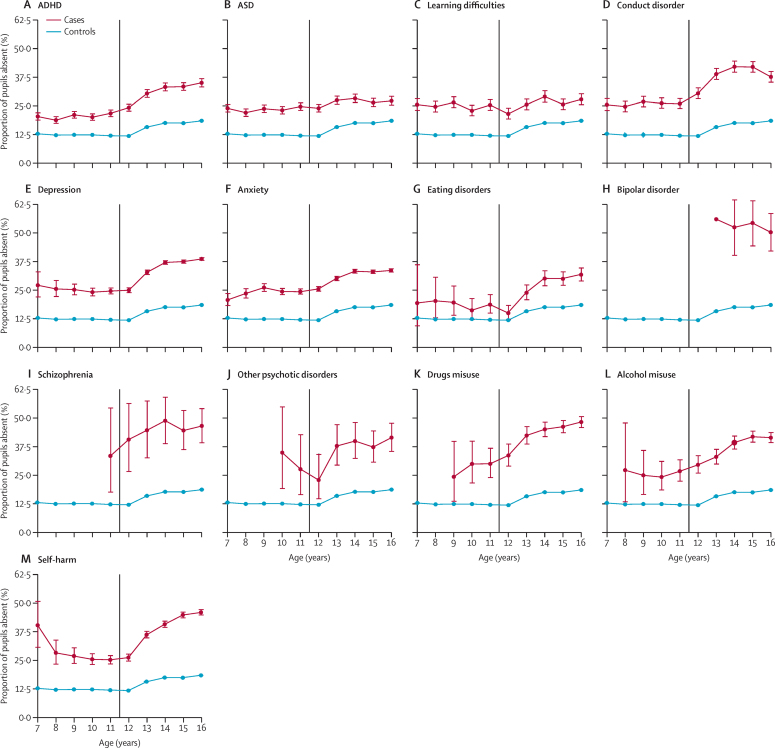
Proportion of absenteeism stratified by diagnosis and age (A) ADHD. (B) ASD. (C) Learning difficulties. (D) Conduct disorder. (E) Depression. (F) Anxiety. (G) Eating disorders. (H) Bipolar disorder. (I) Schizophrenia. (J) Other psychotic disorders. (K) Drugs misuse. (L) Alcohol misuse. (M) Self-harm. Ages represent a birthday during a school year (16th birthday during the last academic year of school). The error bars represent 95% CIs for proportions. The vertical line marks the transition from primary school to secondary school. ASD=autism spectrum disorder.

The proportion of absentee pupils with no record of any of the disorders studied remained stable in primary school (7–11-year-olds) at around 12·5% and increased in secondary school (11–16-year-olds) to around 18% for 16-year-olds (figure 2). For the raw counts used to create figure 2, please see the appendix (pp 16–20). Across all ages, a higher proportion of pupils with a neurodevelopmental disorder, mental disorder, or self-harm record were absent from school compared with pupils without a record (figure 2). In the last 2 years of primary school (10–11-year-olds), pupils with a subsequent diagnosis of schizophrenia or drugs misuse had the highest rate of absenteeism at around 30–33% (figure 2). In the last 2 years of secondary school (ages 15–16 years), pupils with a record of bipolar disorder, schizophrenia, alcohol misuse, drugs misuse, or self-harm had the highest rate of absenteeism at around 40–55% (figure 2).

Goodness-of-fit tests (appendix 2 p 27) showed that including sex, age, and deprivation as covariates sequentially improved the fit, so we present both unadjusted and adjusted results (table 1). Having a record of a neurodevelopmental disorder (OR 2·1, 95% CI 2·0–2·2), mental disorder (2·9, 2·8–2·9), or self-harm (4·0, 3·8–4·1) was associated with absenteeism (table 1). Adjusted ORs (aORs) ranged from 2·0 (95% CI 1·9–2·0) for pupils with a neurodevelopmental disorder to 4·2 (3·4–5·3) for those with schizophrenia and 5·5 (4·2–7·2) for those with bipolar disorder (table 1).

**Table 1:** Main and sensitivity analyses of absenteeism by neurodevelopmental disorder, mental disorder, drugs or alcohol misuse, and self-harm

	**Main analysis**		**Sensitivity analyses**		
	Full cohort (n=414 637; girls=201 789; boys=212 848)		People with one morbidity (n=41 018; girls=21 161; boys=19 857)	People with 2–8 morbidities (n=16 912; girls=9702; boys=7210)	First record at <17 years of age (n=42 734; girls=21 616; boys=21 118)
	OR (95% CI)	aOR (95% CI)*	aOR (95% CI)*	aOR (95% CI)*	aOR (95% CI)*
Neurodevelopmental disorders (ADHD and ASD; n=13 764; girls=2880; boys=10 884)	2·1 (2·0–2·2)	2·0 (1·9–2·0)	..	..	..
ADHD (n=8199; girls=1625; boys=6574)	2·2 (2·1–2·3)	2·0 (1·9–2·0)	1·6 (1·5–1·7)	2·4 (2·3–2·6)	1·9 (1·9–2·0)
ASD (n=7055; girls=1516; boys=5539)	2·2 (2·0–2·2)	2·0 (1·9–2·1)	1·6 (1·5–1·7)	2·5 (2·4–2·7)	1·9 (1·8–2·0)
Learning difficulties (n=3867; girls=1272; boys=2595)	2·1 (2·0–2·2)	2·0 (1·9–2·1)	1·7 (1·6–1·9)	2·3 (2·1–2·6)	1·9 (1·8–2·1)
Conduct disorder (n=4417; girls=1434; boys=2983)	3·0 (2·8–3·1)	2·6 (2·5–2·7)	2·1 (2·0–2·3)	3·1 (2·9–3·3)	2·6 (2·5–2·7)
Any mental disorder (n=37 246; girls=24 328; boys=12 918)	2·9 (2·8–2·9)	2·5 (2·5–2·6)	..	..	..
Depression (n=22 888; girls=15 198; boys=7690)	3·3 (3·2–3·4)	2·8 (2·8–2·9)	2·3 (2·2–2·4)	3·6 (3·5–3·7)	3·1 (3·0–3·2)
Anxiety (n=19 727; girls=13 118; boys=6609)	2·7 (2·6–2·8)	2·5 (2·4–2·5)	1·8 (1·8–1·9)	3·5 (3·4–3·7)	2·5 (2·4–2·5)
Eating disorders (n=1504; girls=1320; boys=184)	2·2 (2·0–2·4)	2·1 (1·9–2·3)	1·2 (1·0–1·4); p=0·064	3·0 (2·7–3·4)	2·2 (2·0–2·4)
Bipolar disorder (n=164; girls=132; boys=32)	6·6 (4·9–8·3)	5·5 (4·2–7·2)	6·1 (2·3–15·7)	5·4 (4·1–7·2)	6·5 (4·0–10·6)
Schizophrenia (n=217; girls=80; boys=137)	5·0 (3·9–6·1)	4·2 (3·4–5·3)	1·8 (0·7–5·0); p=0·25	4·5 (3·6–5·7)	3·9 (2·7–5·7)
Other psychotic disorders (n=327; girls=159; boys=168)	3·6 (3·0–4·4)	3·2 (2·6–3·8)	1·3 (0·7–2·7); p=0·44	3·5 (2·8–4·2)	3·2 (2·4–4·2)
Drugs misuse (n=1990; girls=774; boys=1216)	4·9 (4·6–5·3)	4·1 (3·8–4·4)	3·0 (2·6–3·6)	4·5 (4·1–4·9)	4·6 (4·1–5·1)
Alcohol misuse (n=2434; girls=1313; boys=1121)	3·8 (3·6–4·1)	3·2 (3·0–3·5)	2·2 (2·0–2·5)	4·3 (4·0–4·7)	3·7 (3·4–4·0)
Self-harm (n=8706; girls=6652; boys=2054)	4·0 (3·8–4·1)	3·4 (3·3–3·6)	2·7 (2·5–2·8)	3·9 (3·7–4·1)	3·5 (3·4–3·7)

All results are highly significant (p<0·0001), unless otherwise specified. Neurodevelopmental disorders comprise ADHD and ASD. The category of any mental disorders comprises depression, anxiety, eating disorders, schizophrenia, bipolar disorder, and other psychotic disorders. aOR=adjusted odds ratio. ASD=autism spectrum disorder. OR=odds ratio.

* Adjusted for sex, age, and deprivation.

Of those with a record of neurodevelopmental disorders, learning difficulties, conduct disorder, depression, other psychotic disorders, or drugs or alcohol misuse, boys were less likely to be absent than were girls (appendix 2 p 9). For those with a record of anxiety, eating disorders, bipolar disorder, schizophrenia, or self-harm, sex was not significantly associated with absenteeism (appendix 2 p 9). For pupils with a record of neurodevelopmental disorders, conduct disorder, depression, anxiety, eating disorders, drugs or alcohol misuse, or self-harm, age was associated with absenteeism, with slight increases in ORs per year (appendix 2 p 9). The sample sizes for bipolar disorder and schizophrenia were too small to assess the association of deprivation quintile with absenteeism; however, the odds of being absent increased with increased deprivation (5th *vs* 1st quintile) for all other variables apart from other psychotic disorders, ranging from 1·5 (95% CI 1·3–1·9) for conduct disorder to 2·8 (2·2–3·6) for alcohol misuse (appendix 2 p 9).

**Figure 3:**
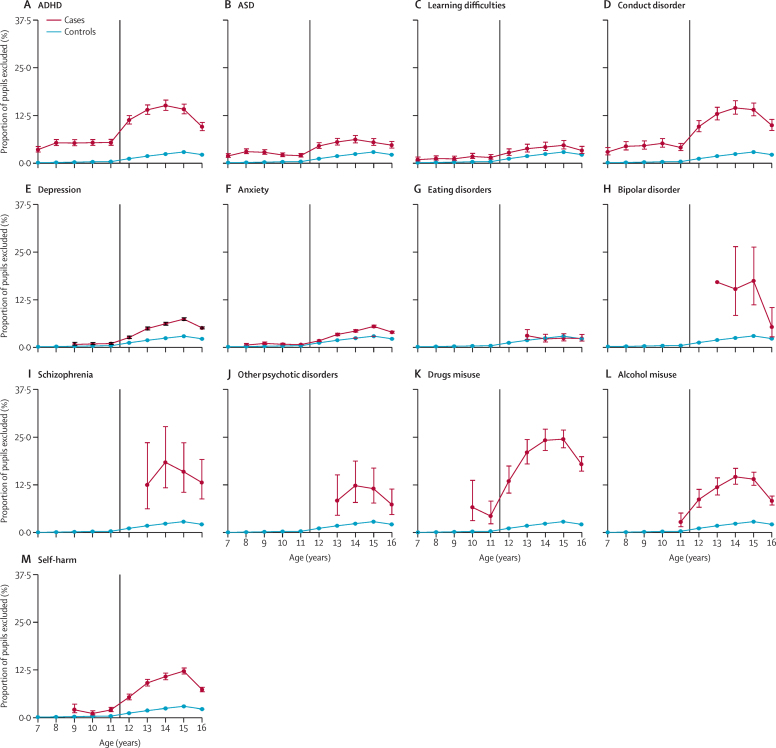
Proportion of exclusions stratified by diagnosis and age (A) ADHD. (B) ASD. (C) Learning difficulties. (D) Conduct disorder. (E) Depression. (F) Anxiety. (G) Eating disorders. (H) Bipolar disorder. (I) Schizophrenia. (J) Other psychotic disorders. (K) Drugs misuse. (L) Alcohol misuse. (M) Self-harm. Ages represent a birthday during a school year (16th birthday during the last academic year of school). The error bars represent 95% CIs for proportions. The vertical line marks the transition from primary school to secondary school. ASD=autism spectrum disorder.

15 199 (3·7%) of 414 637 pupils had been excluded from school at least once, 243 (0·1%) of whom were excluded permanently. 1979 (13·0%) of 15 199 had a neurodevelopmental disorder, 3161 (20·8%) had a mental disorder, and 1518 (10·0%) had a record of self-harm. 4568 (30·1%) were diagnosed while of school age, of whom 1925 (42·1%) had a neurodevelopmental disorder, 2048 (44·8%) had a mental disorder, and 1291 (28·3%) had a record of self-harm. Children aged 7–11 years with no record of the studied diagnoses or self-harm were very unlikely to be excluded (1174 [0·5%] of 233 191). They were more likely to be excluded if they had a record of ASD (211 [4·7%] of 4464) or conduct disorder (193 [8·0%] of 2415). Exclusions generally became more common among older children (figure 3). For those with no disorder or self-harm, the proportion of exclusions increased to 2·9% (2770 of 95 977) among those aged 15 years, before decreasing to 2·2% (2064 of 94 172) in the last year of secondary school (age 16 years). Notable increases in exclusion rates were seen among pupils aged 14 years with ADHD (374 [15·1%] of 2483), conduct disorder (207 [14·5%] of 1433), drugs misuse (205 [24·2%] of 848), alcohol misuse (150 [14·6%] of 1026), and self-harm (443 [10·7%] 4135), although exclusion rates tended to decrease in the final year of secondary school (figure 3). The proportion of pupils with severe mental illness who were excluded was also high, with 16 (17·4%) of 92 with bipolar disorder excluded at age 15 years and 16 (18·4%) of 87 with schizophrenia excluded at age 14 years (figure 3). For the raw counts used to create figure 3, please see the appendix (pp 21–24).

Goodness-of-fit tests (appendix 2 p 27) again showed that including sex, age, and deprivation as covariates sequentially improved model fit. Having a neurodevelopmental disorder, a mental disorder, or a record of self-harm were all associated with being excluded from school (table 2). After adjusting for sex, age, and deprivation, pupils with a record of drugs misuse had the highest odds of being excluded (table 2). To note, alcohol misuse, self-harm, schizophrenia, and bipolar disorder also had high aORs (table 2).

**Table 2:** Main and sensitivity analyses of exclusion by neurodevelopmental disorder, mental disorder, drugs or alcohol misuse, and self-harm

	**Main analysis**		**Sensitivity analyses**		
	Full cohort (n=414 637; girls=201 789; boys=212 848)		People with one morbidity (n=41 018; girls=21 161; boys=19 857)	People with 2–8 morbidities (n=16 912; girls=9702; boys=7210)	First record at <17 years of age (n=42 734; girls=21 616; boys=21 118)
	OR (95% CI)	aOR (95% CI)*	aOR (95% CI)*	aOR (95% CI)*	aOR (95% CI)*
Neurodevelopmental disorders (ADHD and ASD; n=13 764; girls=2880; boys=10 884)	6·3 (6·0–6·6)	4·4 (4·2–4·6)	..	..	..
ADHD (n=8199; girls=1625; boys=6574)	8·9 (8·4–9·5)	6·1 (5·7–6·4)	5·0 (4·6–5·5)	7·2 (6·7–7·8)	6·0 (5·7–6·4)
ASD (n=7055; girls=1516; boys=5539)	3·6 (3·3–3·9)	2·6 (2·4–2·9)	1·7 (1·4–1·9)	3·5 (3·2–4·0)	2·6 (2·4–2·9)
Learning difficulties (n=3867; girls=1272; boys=2595)	2·4 (2·1–2·7)	1·8 (1·5–2·0)	1·0 (0·8–1·3); p=0·95	2·7 (2·3–3·2)	1·7 (1·5–2·0)
Conduct disorder (n=4417; girls=1434; boys=2983)	8·6 (7·9–9·3)	6·0 (5·5–6·5)	4·1 (3·6–4·7)	7·7 (6·9–8·5)	6·0 (5·5–6·5)
Any mental disorder (n=37 246; girls=24 328; boys=12 918)	4·0 (3·8–4·2)	2·8 (2·7–3·0)	..	..	..
Depression (n=22 888; girls=15 198; boys=7690)	5·0 (4·7–5·2)	3·3 (3·1–3·4)	2·1 (1·9–2·2)	4·7 (4·4–5·0)	3·2 (3·0–3·5)
Anxiety (n=19 727; girls=13 118; boys=6609)	3·3 (3·1–3·5)	2·4 (2·3–2·6)	1·2 (1·1–1·3)	4·1 (3·8–4·4)	2·0 (1·8–2·2)
Eating disorders (n=1504; girls=1320; boys=184)	1·7 (1·3–2·2)	1·9 (1·5–2·4)	1·1 (0·6–1·8); p=0·78	2·5 (1·8–3·3)	2·2 (1·7–2·8)
Bipolar disorder (n=164; girls=132; boys=32)	10·5 (7·2–15·3)	7·3 (4·9–10·9)	5·1 (0·8–30·7); p=0·075	7·4 (4·9–11·2)	6·8 (3·4–13·8)
Schizophrenia (n=217; girls=80; boys=137)	14·3 (10·4–19·6)	6·5 (4·6–9·2)	4·7 (1·5–14·6); p=0·0068	6·7 (4·7–9·7)	3·1 (1·6–5·8)
Other psychotic disorders (n=327; girls=159; boys=168)	7·6 (5·5–10·6)	4·2 (3·0–5·9)	1·4 (0·4–4·5); p=0·56	4·6 (3·2–6·6)	2·9 (1·5–5·5)
Drugs misuse (n=1990; girls=774; boys=1216)	22·7 (20·8–24·7)	11·0 (10·0–12·1)	8·4 (7·0–10·2)	11·9 (10·7–13·2)	14·2 (12·5–16·1)
Alcohol misuse (n=2434; girls=1313; boys=1121)	11·1 (10·1–12·3)	6·3 (5·7–7·0)	3·3 (2·7–4·0)	9·3 (8·2–10·6)	8·1 (7·2–9·2)
Self-harm (n=8706; girls=6652; boys=2054)	8·2 (7·7–8·7)	6·7 (6·3–7·2)	4·8 (4·2–5·4)	7·7 (7·1–8·3)	7·3 (6·8–7·9)

All results are highly significant (p<0·0001), unless otherwise specified. Neurodevelopmental disorders comprise ADHD and ASD. The category of any mental disorders comprises depression, anxiety, eating disorders, schizophrenia, bipolar disorder, and other psychotic disorders. aOR=adjusted odds ratio. ASD=autism spectrum disorder. OR=odds ratio.

* Adjusted for sex, age, and deprivation.

Across disorders, apart from bipolar disorder, boys were significantly more likely to be excluded than were girls (appendix 2 p 13). Boys with a record of learning difficulties, anxiety, eating disorders, schizophrenia, other psychotic disorders, or self-harm had an OR for being excluded between 2 and 3 (appendix 2 p 13). Being older was associated with a higher odds of exclusion for individuals with a record of most variables studied (OR range 1·09–1·19), except for bipolar disorder, schizophrenia, other psychotic disorders, drugs misuse, and alcohol misuse (appendix 2 p 13). The sample sizes for bipolar disorder and schizophrenia were too small to assess the association of deprivation quintile with exclusion; however, the odds of exclusion were higher in the most deprived areas than in the least deprived areas for all variables apart from other psychotic disorders, with the OR varying from 1·4 (95% CI 1·1–1·9) for those with conduct disorder to 3·3 (2·7–4·0) for those with anxiety (appendix 2 p 13).

Pupils in our cohort had up to eight morbidities in total. 41 018 had one morbidity, 12 096 had two, 3495 had three, and 1321 had four or more (appendix 2 p 10). Absenteeism was more likely in pupils with comorbidities than in pupils with one morbidity, except in the case of bipolar disorder (table 1). Pupils with a single diagnosis of an eating disorder, schizophrenia, or other psychotic disorder were not at higher risk of being absent compared with their healthy peers (table 1). When the number of comorbidities was modelled as a covariate, the OR of being absent was between 1·2 and 1·4 for each additional comorbidity, except for bipolar disorder for which the OR was 1·0 (appendix 2 p 11). SEN status did not reduce the ORs for being absent in those with anxiety, eating disorders, schizophrenia, or alcohol misuse (appendix 2 p 12). For those with ADHD, ASD, learning difficulties, conduct disorder, depression, bipolar disorder, drugs misuse, other psychotic disorders, or a record of self-harm, having SEN status reduced the OR for absenteeism to 0·59–0·89 compared with not having SEN status (appendix 2 p 12). The results for pupils with a record before 17 years of age were similar to those of the main cohort, except for pupils with a record of alcohol or drugs misuse, bipolar disorder, or depression who had slightly higher odds of being absent, and for pupils with schizophrenia who had slightly lower odds (table 1).

In the group of pupils with more than one morbidity, aORs for being excluded were consistently higher than for those with one morbidity (table 2). When the number of comorbidities was modelled as a covariate, the OR of being excluded was between 1·2 and 1·8 per each additional comorbidity (appendix 2 p 14). SEN status was associated with decreasing ORs for being excluded for those with neurodevelopmental disorders, conduct disorder, depression, bipolar disorder, other psychotic disorders, drugs misuse, and self-harm (appendix 2 p 15). aORs for being excluded differed between the subpopulation diagnosed while at school and the main cohort, depending on the variable (table 2). Of note, there was little difference for neurodevelopmental disorders, the aOR for exclusion was lower for people with schizophrenia when diagnosed while at school, and the aORs for exclusion were somewhat higher for individuals with drugs or alcohol misuse recorded while of school age (table 2).

The individual-level yearly absence rate (number of sessions missed/total number of possible sessions per year) was higher in the group with a record of exclusion than in the group without a record of exclusion (appendix 2 p 5). The correlation between individual-level yearly absence rates and individual-level yearly exclusion rates was *r*=0·17 in the full cohort and *r*=0·35 for those with recorded exclusions.

## Discussion

Our study, which involved more than 400 000 pupils, highlights that children and young people diagnosed with a neurodevelopmental disorder or mental disorder, or who have a record of self-harm, before 24 years of age are much more likely to miss school than their peers, even after adjusting for age, sex, and deprivation. Our data and study size enabled us to include disorders typically not included in studies of school-aged children, such as rare disorders and disorders that typically present after individuals have left school (eg, schizophrenia), that might confer antecedent clinical vulnerabilities.^32^ School absenteeism and exclusion rates were higher after 11 years of age for all children but disproportionally more so in those with a record of a disorder or self-harm, even if it was recorded during school age. This finding could reflect a reduced direct influence of parents on older children's attendance or the smaller size of primary schools compared with secondary schools. Generally, individuals with more than one recorded morbidity were more likely to be absent or excluded than were those with only one morbidity, which was exacerbated with each additional disorder. Within the diagnosed populations, girls with neurodevelopmental disorders, learning difficulties, conduct disorder, depression, other psychotic disorders, or drugs or alcohol misuse were more likely to be absent than were boys, and boys were more likely to be excluded than were girls across all studied disorders apart from bipolar disorder. This finding aligns with the view that boys externalise mental distress through their behaviour, which in turn impacts the school environment and results in their exclusion, whereas girls, and especially those with emotional disorders or delayed diagnosis of neurodevelopmental disorders, tend to be more anxious and withdraw from social contact.^32^ Age was found to be associated with both outcomes in relation to most disorders. We also found associations between both outcomes and deprivation within most disorders studied. Having SEN status reduced the likelihood of being absent or excluded, most notably for those with records of neurodevelopmental disorders or bipolar disorder, compared with those with a record but no SEN status, potentially highlighting the positive impact of recognition, diagnosis, and educational interventions.

Our findings strengthen those found previously in much smaller population-based studies. In the ALSPAC study^29^ of a UK birth cohort, by 8 years of age, 19% of children with ADHD and 31% of those with conduct disorder were excluded from school compared with 1·9% and 2·8% of those without ADHD or conduct disorder, respectively. In another study of a UK cohort (BCAMHS),^30^ psychiatric symptoms (assessed through validated questionnaires) were a significant predictor of exclusions.

Our study is based on routinely collected data encompassing a wide range of clinically diagnosed and recorded disorders. It benefits from well documented, often validated, and curated lists of ICD-10 and read (version 2) codes to ascertain each of the disorders. Arguably, diagnoses made by clinicians for those in contact with services provide more complete case ascertainment than do surveys or cohort studies, which are susceptible to selection bias due to low recruitment and high attrition in populations with psychiatric disorders. However, a common feature of all database studies of routinely collected data is the underestimation of the number of disorders in the population as not all those affected consult their GP, or conditions might not be recognised or recorded.^33^ Additionally, there is no validated measure of the clinical problems recorded, which prevents any estimation of severity, and administrative data are vulnerable to random errors in data entry.

This study's novelty lies in its linkage of education, health (including primary care), and deprivation datasets for a whole population (Wales) at an individual pupil level over 4 school years for a wide range of disorders. Linking health and education data on this scale allows us to gain valuable insights on the education of children with neurodevelopmental disorders, mental health disorders, or self-harm. Because many older adolescents with common mental disorders are managed in primary care, it is important to include this data source. A whole population dataset enabled us to include pupils with rarer conditions such as schizophrenia and bipolar disorder. Linking diagnoses up to the age of 24 years allowed for assessment of conditions more frequently diagnosed after school leaving age (eg, schizophrenia), for which their antecedents or premorbid presentation, such as cognitive or social deficits, apathy, or self-medication with drugs, might affect attendance and exclusion. We did not take physical comorbidities into account, although we note the strong association between poor mental and physical health,^34^ because some absences would have been due to physical morbidity and medication rather than the mental or neurodevelopmental disorder, which would have complicated the interpretation of our findings.

Our estimates might underestimate the effect of mental health difficulties on exclusions and absenteeism. Younger children will have had less time for evidence of their diagnosis to be recorded, especially for those conditions that tend to appear later in adolescence, and some young people who are diagnosable will not present to services. There is some evidence^30^ to suggest that, for each diagnosed child, there could be a number that have multiple symptoms but do not meet the criteria for a diagnosis. These children might well have issues at school that could lead to poor attendance or exclusion. Some children, especially those with ADHD, ASD, or learning difficulties, might not have been included in our dataset because they are in schools for children with special educational or behavioural needs or are home-schooled. Different pupils contributed different numbers of years to the analysis. We are satisfied that there were no demographic or mental health-related differences between these pupils. 5·2% of pupils with education data in the 2013–16 academic years did not have any linked health data and were removed from our analyses.

There are various processes through which school attendance might be associated with neurodevelopmental disorders, mental disorders, and self-harm. These processes include disruptive behaviours resulting in exclusion, physical comorbidities or somatic symptoms (eg, stomach pain and headaches) leading to authorised absence, symptoms associated with anxiety and depression leading to school refusal, family problems, and peer problems (eg, bullying). If absence from school results in social isolation and poorer academic performance, it could exacerbate mental health and attendance issues if the cycle is not disrupted. Our study cannot infer causal relationships and further research should focus on the direction of the association, which could be bidirectional for individual disorders and outcomes. Clinical record data might not be ideal to use in these future studies because the documentation in clinical records will not represent an accurate measure of the time of first onset of the symptoms or disorder. However, even without an understanding of the direction or mechanisms of the association, the demonstration of an association using real life outcomes and data is important.

Poor school attendance affects the educational attainment of children and future social, developmental, employment, and physical health outcomes. Many governments, including UK Governments, have recognised the importance of regular school attendance and have issued related guidelines, which include penalty notices for the carers or parents of persistently absent children and the use of incentives to encourage high attendance.^35^ Exclusions from schools in England and Wales are intended to be used in serious breaches of behaviour policies—for violence, sexual abuse, the supply of illegal drugs, or the use of weapons.^36^, ^37^ Currently, rates of exclusion in England are rising, raising concerns about school-based policies to improve behaviour and support teachers. Similar initiatives are in place in the USA and elsewhere.^38^

Linking routinely collected health and education data has the potential to improve services for children^39^ by identifying those in need, alongside gaps in provision. Our analysis clearly shows that children with neurodevelopmental disorders, mental disorders, and self-harm spend less time at school. As such, exclusion or persistent absence is a potential indicator for current or future poor mental health that is routinely collected by schools and local education authorities and could be used to target assessment and early intervention.^40^ There is growing interest in school-based prevention and early intervention programmes that focus on improving the school climate for reducing adolescent mental health problems,^41^, ^42^ which has relevance now as children return to school following closures and blended learning in response to the COVID-19 pandemic. Other interventions have included psychological interventions that focus primarily on anxiety and depression symptoms.^43^ School-based mental health provision and integration with mental health services has been highlighted as a major strategic priority in the UK.^44^ This approach could benefit young people, as supported by our finding that having a SEN status decreases the odds of being absent or excluded, even if it does not remove the risk completely. Attendance and exclusion data, which are already collected by schools, could provide useful information about where to focus sparse resources. School-based mental health prevention strategies might also help to build resilience, enabling pupils to develop strategies for managing and improving their mental health and wellbeing, and to understand when and how to seek additional help.

Future research could further explore whether improvements in school attendance over time serve to reduce the incidence of mental disorders and whether the timing of diagnosis is an important factor in the risk for absenteeism or exclusions. This can be done by looking at causal relationships between mental health and school outcomes using a longer follow-up period. Other avenues for research include evaluating the effect of physical comorbidities on school outcomes and the differential associations of pairs of disorders with school outcomes.

To conclude, people up to 24 years of age who have mental or neurodevelopmental disorders or self-harm have poorer attendance at school than their peers who do not have disorders or self-harm. Exclusion or persistent absence is a potential indicator of current or future poor mental health that is routinely collected by schools and local education authorities and could be used to target assessment and early intervention.

## Data sharing

The data used in this study are available in the SAIL Databank at Swansea University (Swansea, UK) via the Adolescent Mental Health Data Platform, but, as restrictions apply, they are not publicly available. All proposals to use SAIL data are subject to review by an independent Information Governance Review Panel. Before any data can be accessed, approval must be given by the Information Governance Review Panel. The Information Governance Review Panel carefully considers each project to ensure proper and appropriate use of SAIL data. When access has been granted, it is gained through a privacy-protecting safe haven and remote access system referred to as the SAIL Gateway. SAIL have established an application process to be followed by anyone who would like to access data via SAIL, details of which can be found at https://www.saildatabank.com/application-process. Derived data supporting the findings of this study are available from the corresponding author (AJ) on request at a.john@swansea.ac.uk.

## Declaration of interests

AJ is a trustee of the Samaritans. All other authors declare no competing interests.

## Supplementary Materials

Welsh translation of the abstract

## References

[1] Aucejo EM, Romano TF (2016). Assessing the effect of school days and absences on test score performance. Econ Educ Rev.

[2] Hancock KJ, Lawrence D, Shepherd CCJ, Mitrou F, Zubrick SR (2017). Associations between school absence and academic achievement: do socioeconomics matter?. Br Educ Res J.

[3] Reid K (2002).

[4] Henry KL, Huizinga DH (2007). Truancy's effect on the onset of drug use among urban adolescents placed at risk. J Adolesc Health.

[5] Vaughn MG, Wexler J, Beaver KM, Perron BE, Roberts G, Fu Q (2011). Psychiatric correlates of behavioral indicators of school disengagement in the United States. Psychiatr Q.

[6] Berg I, Butler A, Franklin J, Hayes H, Lucas C, Sims R (1993). DSM-III-R disorders, social factors and management of school attendance problems in the normal population. J Child Psychol Psychiatry.

[7] Bools C, Foster J, Brown I, Berg I (1990). The identification of psychiatric disorders in children who fail to attend school: a cluster analysis of a non-clinical population. Psychol Med.

[8] Finning K, Ukoumunne OC, Ford T (2019). Review: the association between anxiety and poor attendance at school—a systematic review. Child Adolesc Ment Health.

[9] Lawrence D, Dawson V, Houghton S, Goodsell B, Sawyer MG (2019). Impact of mental disorders on attendance at school. Aust J Educ.

[10] McShane G, Walter G, Rey JM (2001). Characteristics of adolescents with school refusal. Aust N Z J Psychiatry.

[11] Lereya ST, Patel M, Dos Santos JPGA, Deighton J (2019). Mental health difficulties, attainment and attendance: a cross-sectional study. Eur Child Adolesc Psychiatry.

[12] Epstein S, Roberts E, Sedgwick R (2020). School absenteeism as a risk factor for self-harm and suicidal ideation in children and adolescents: a systematic review and meta-analysis. Eur Child Adolesc Psychiatry.

[13] Fleming M, Fitton CA, Steiner MFC (2017). Educational and health outcomes of children treated for attention-deficit/hyperactivity disorder. JAMA Pediatr.

[14] Black LI, Zablotsky B (2018). Chronic school absenteeism among children with selected developmental disabilities: national health interview survey, 2014–2016. Natl Health Stat Rep.

[15] Parker C, Whear R, Ukoumunne OC (2015). School exclusion in children with psychiatric disorder or impairing psychopathology: a systematic review. Emot Behav Difficulties.

[16] Fröjd SA, Kaltiala-Heino R, Marttunen MJ (2011). Does problem behaviour affect attrition from a cohort study on adolescent mental health?. Eur J Public Health.

[17] Wolke D, Waylen A, Samara M (2009). Selective drop-out in longitudinal studies and non-biased prediction of behaviour disorders. Br J Psychiatry.

[18] Saiepour N, Ware R, Najman J, Baker P, Clavarino A, Williams G (2016). Do participants with different patterns of loss to follow-up have different characteristics? A multi-wave longitudinal study. J Epidemiol.

[19] Ford DV, Jones KH, Verplancke JP (2009). The SAIL Databank: building a national architecture for e-health research and evaluation. BMC Health Serv Res.

[20] Lyons RA, Jones KH, John G (2009). The SAIL databank: linking multiple health and social care datasets. BMC Med Inform Decis Mak.

[21] Sadler K, Vizard T, Ford T (Nov 22, 2018). Mental health of children and young people in England, 2017. https://digital.nhs.uk/data-and-information/publications/statistical/mental-health-of-children-and-young-people-in-england/2017/2017.

[22] NHS Digital (2018). Read codes. https://digital.nhs.uk/services/terminology-and-classifications/read-codes.

[23] Estyn (June 12, 2015). Effective practice in improving attendance in primary schools—June 2015. https://www.estyn.gov.wales/thematic-report/effective-practice-improving-attendance-primary-schools-june-2015.

[24] Estyn (Sept 1, 2014). Attendance in secondary schools—September 2014. https://www.estyn.gov.wales/thematic-report/attendance-secondary-schools-september-2014.

[25] Department of Education (March, 2019). A guide to absence statistics. https://assets.publishing.service.gov.uk/government/uploads/system/uploads/attachment_data/file/787314/Guide_to_absence_statistics_21032019.pdf.

[26] Welsh Government (Aug 29, 2019). Absenteeism from secondary schools, 2018/19. https://gov.wales/sites/default/files/statistics-and-research/2019-08/absenteeism-from-secondary-schools-september-2018-august-2019-318.pdf.

[27] Højsgaard S, Halekoh U, Yan J (2006). The R package geepack for generalized estimating equations. J Stat Softw.

[28] Liang KY, Zeger SL (1993). Regression analysis for correlated data. Annu Rev Public Health.

[29] Paget A, Parker C, Heron J (2018). Which children and young people are excluded from school? Findings from a large British birth cohort study, the Avon Longitudinal Study of Parents and Children (ALSPAC). Child Care Health Dev.

[30] Ford T, Parker C, Salim J, Goodman R, Logan S, Henley W (2018). The relationship between exclusion from school and mental health: a secondary analysis of the British Child and Adolescent Mental Health Surveys 2004 and 2007. Psychol Med.

[31] Pan W (2001). Akaike's information criterion in generalized estimating equations. Biometrics.

[32] Pine DS, Fox NA (2015). Childhood antecedents and risk for adult mental disorders. Annu Rev Pyschol.

[33] John A, Marchant AL, Fone DL (2016). Recent trends in primary-care antidepressant prescribing to children and young people: an e-cohort study. Psychol Med.

[34] van der Lee JH, Mokkink LB, Grootenhuis MA, Heymans HS, Offringa M (2007). Definitions and measurement of chronic health conditions in childhood: a systematic review. JAMA.

[35] Welsh Assembly Government (2011). Strategies for schools to improve attendance and manage lateness. https://dera.ioe.ac.uk/2945/3/110308section3en.pdf.

[36] Department for Education (September, 2017). Exclusion from maintained schools, academies and pupil referral units in England. https://assets.publishing.service.gov.uk/government/uploads/system/uploads/attachment_data/file/641418/20170831_Exclusion_Stat_guidance_Web_version.pdf.

[37] Welsh Government (April 1, 2015). Exclusions from schools and pupil referral units (PRU). https://gov.wales/exclusion-schools-and-pupil-referral-units-pru.

[38] US Department of Education (Oct 7, 2015). Key policy letters signed by the Education Secretary or Deputy Secretary. https://www2.ed.gov/policy/elsec/guid/secletter/151007.html.

[39] Downs J, Gilbert R, Hayes RD, Hotopf M, Ford T (2017). Linking health and education data to plan and evaluate services for children. Arch Dis Child.

[40] Kearney CA, Graczyk P (2014). A response to intervention model to promote school attendance and decrease school absenteeism. Child Youth Care Forum.

[41] Shinde S, Weiss HA, Varghese B (2018). Promoting school climate and health outcomes with the SEHER multi-component secondary school intervention in Bihar, India: a cluster-randomised controlled trial. Lancet.

[42] Bonell C, Blakemore S-J, Fletcher A, Patton G (2019). Role theory of schools and adolescent health. Lancet Child Adolesc Health.

[43] Fleming T, Dixon R, Frampton C, Merry S (2012). A pragmatic randomized controlled trial of computerized CBT (SPARX) for symptoms of depression among adolescents excluded from mainstream education. Behav Cogn Psychother.

[44] Department of Health & Social Care, Department for Education (December, 2017). Transforming children and young people's mental health provision: a green paper. https://assets.publishing.service.gov.uk/government/uploads/system/uploads/attachment_data/file/664855/Transforming_children_and_young_people_s_mental_health_provision.pdf.

