# Supplementary material for: Association of school absence and exclusion with recorded neurodevelopmental disorders, mental disorders, or self-harm: a nationwide, retrospective, electronic cohort study of children and young people in Wales, UK
Source: Lancet Psychiatry. 2022 Jan;9(1):23–34. doi: 10.1016/S2215-0366(21)00367-9 (PMC8674147; doi:10.1016/S2215-0366(21)00367-9)
Supplement: Welsh translation of the abstract [file mmc1.pdf]

THE LANCET
Psychiatry

Supplementary appendix 1

This translation in Welsh was submitted by the authors and we reproduce it as supplied. It has not been peer reviewed. *The Lancet*'s editorial processes have only been applied to the original in English, which should serve as reference for this manuscript.

Cyflwynwyd y cyfieithiad Cymraeg hwn gan yr awduron a rydym yn ei atgynhyrchu yma yn yr un ffurf. Ni chafwyd ei adolygu gan gymheiriaid. Dim ond y gwreddiol yn Saesneg sydd wedi ei brosesu'n golgyddol gan Y *Lancet*, a hwn dylai fod y cyfeirnod i'r llawysgrif hon.

Supplement to: John A, Friedmann Y, DelPozo-Banos M, et al. Association of school absence and exclusion with recorded neurodevelopmental disorders, mental disorders, or self-harm: a nationwide, retrospective, electronic cohort study of children and young people in Wales, UK. *Lancet Psychiatry* 2021; published online Nov 23. http://dx.doi.org/10.1016/S2215-0366(21)00367-9.

Crynodeb

Cefndir Presenoldeb gwael yn yr ysgol, pa un ai a ydy oherwydd absenolrwydd neu waharddiad, yn arwain at anfanteision cymdeithasol lluosog, addysgol, a hir-oes economaidd gymdeithasol. Ein bwriad oedd mesur y cydgysylltiad rhwng ystod eang o anhwylderau niwroddatblygiadol a meddyliol wedi'u diagnosio a hunan-niwed wedi eu cofnodi erbyn 24 oed a phresenoldeb ysgol a gwaharddiad.

Dulliau Yn yr astudiaeth garfan electronig ôl-weithredol, genedlaethol hon, gwnaethom dynnu carfan o Set Ddata Gwasanaeth Demograffig Cymru, wnaeth gynnwys unigolion 7-16 mlwydd oed (16 oed yw'r oedran gadael ysgol yn y DU) wedi cofrestru mewn ysgolion a ariennir gan y wladwriaeth yng Nghymru ym mlynyddoedd academaidd 2012/13-2015/16 (rhwng Medi 1, 2012, ac Awst 31, 2016).

Gan ddefnyddio Llwyfan Data Iechyd Meddwl y Glasoed, fe wnaethom gysylltu data presenoldeb a gwahardd â setiau data gofal iechyd demograffig a sylfaenol ac eilaidd cenedlaethol. Fe wnaethom nodi pob disgybl a oedd wedi derbyn diagnosis wedi'i gofnodi o anhwylderau niwroddatblygiadol (ADHD ac anhwylder sbectrwm awtistiaeth [ASD]), anawsterau dysgu, anhwylder ymddygiad, gofid, anhwylder bwyta, camddefnydd o alcohol neu gyffuriau, anhwylder deubegwn, sgitsoffrenia, anhwylderau seicotig eraill, neu hunan-niweidio wedi ei gofnodi (ein newidynnau esboniadol) cyn 24 mlwydd oed. Y canlyniadau oedd absenoldeb a gwaharddiad o'r ysgol. Defnyddiwyd hafaliadau amcangyfrif cyffredinol gyda strwythurau cydberthynas cyfnewidiadwy gan ddefnyddio dosbarthiad binomaidd gyda'r swyddogaeth gyswllt logit i gyfrifo cymarebau gwahanol (OR) ar gyfer absenolrwydd a gwaharddiad, gan addasu ar gyfer rhyw, oed, ac amddifadedd.

Darganfyddiadau Roedd data presenoldeb ysgol, gwaharddiad ysgol, a gofal iechyd ar gael ar gyfer 414 637 o ddisgyblion - merched (201 789 [48·7%] a bechgyn 212 848 [51·3%]; oedran cymedrig 10·5 oed (SD 3·8) ar Fedi 1, 2012; (nid oedd data ethnigrwydd ar gael).

Roedd unigolion gyda chofnod o anhwylder niwroddatblygiadol, anhwylder meddwl, neu hunan-niweidio yn fwy tebygol o fod yn absennol neu wedi eu gwahardd mewn unrhyw flwyddyn ysgol na'r rheiny heb gofnod. Roedd ORs heb eu haddasu ar gyfer absenoldebau yn amrywio o 2·1 (95% CI 2·0-2·2) ar gyfer y rheiny gydag anhwylderau niwroddatblygiadol i 6·6 (4·9-8·3) ar gyfer y rheiny ag anhwylder deubegwn.

Roedd ORs wedi'u haddasu (aORs) ar gyfer absenoldebau yn amrywio o 2 · 0 (1 · 9-2 · 1) ar gyfer y rhai ag anhwylderau niwroddatblygiadol i 5·5 (4·2-7·2) i'r rheiny gydag anhwylder deubegwn. Roedd ORs heb eu haddasu i'w gwahardd yn amrywio o 1·7 (1·3-2·2) i'r rhai ag anhwylderau bwyta i 22·7 (20·8-24·7) ar gyfer y rheiny gyda chofnod o gamddefnyddio cyffuriau. Roedd ORs i'w gwahardd yn amrywio o 1·8 (1·5-2·0) i'r rhai ag anawsterau dysgu i 11·0 (10·0-12·1) ar gyfer y rheiny gyda chofnod o gamddefnyddio cyffuriau.

Dehongliad Mae plant a phobl ifanc fyny hyd at 24 mlwydd oed gyda chofnod niwroddatblygiadol neu anhwylder iechyd meddwl neu hunan-niwed cyn 24 oed yn fwy tebygol o golli ysgol na'r rheiny heb gofnod. Mae gwaharddiad neu absenoldeb parhaus yn arwyddion posib o iechyd meddwl gwael nawr neu yn y dyfodol sy'n cael eu casglu fel mater o drefn ac y gellid eu defnyddio i dargedu asesu ac ymyrraeth gynnar. Mae angen strategaethau integredig yn yr ysgol a gofal iechyd i gefnogi ymgysylltiad pobl ifanc â bywyd ysgol.
