# Supplementary material for: Association of school absence and exclusion with recorded neurodevelopmental disorders, mental disorders, or self-harm: a nationwide, retrospective, electronic cohort study of children and young people in Wales, UK
Source: Lancet Psychiatry. 2022 Jan;9(1):23–34. doi: 10.1016/S2215-0366(21)00367-9 (PMC8674147; doi:10.1016/S2215-0366(21)00367-9)
Supplement: Supplementary appendix 2 [file mmc2.pdf]

THE LANCET
Psychiatry

Supplementary appendix 2

This appendix formed part of the original submission and has been peer reviewed. We post it as supplied by the authors.

Supplement to: John A, Friedmann Y, DelPozo-Banos M, et al. Association of school absence and exclusion with recorded neurodevelopmental disorders, mental disorders, or self-harm: a nationwide, retrospective, electronic cohort study of children and young people in Wales, UK. *Lancet Psychiatry* 2021; published online Nov 23. http://dx.doi.org/10.1016/S2215-0366(21)00367-9.

Appendix

Supplementary figure 1: Comparison of the sex distribution in those with missing GP data and those with GP data (n=437,412).

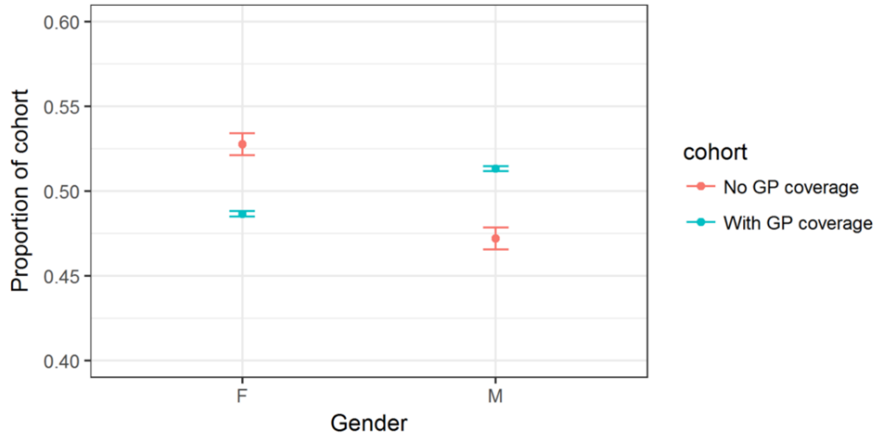

Supplementary figure 2: Comparison of the deprivation distribution in two groups in with missing GP data and those with GP data. Proportion for each group by deprivation quintiles is presented, with quintile 5 being most deprived.

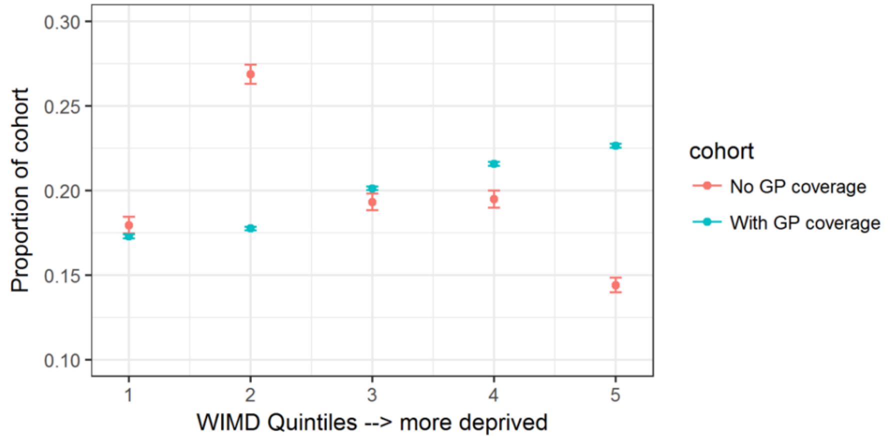

Supplementary figure 3: Proportion of our cohort population with and without a mental disorder (MH), by the number of years contributed to the analysis.

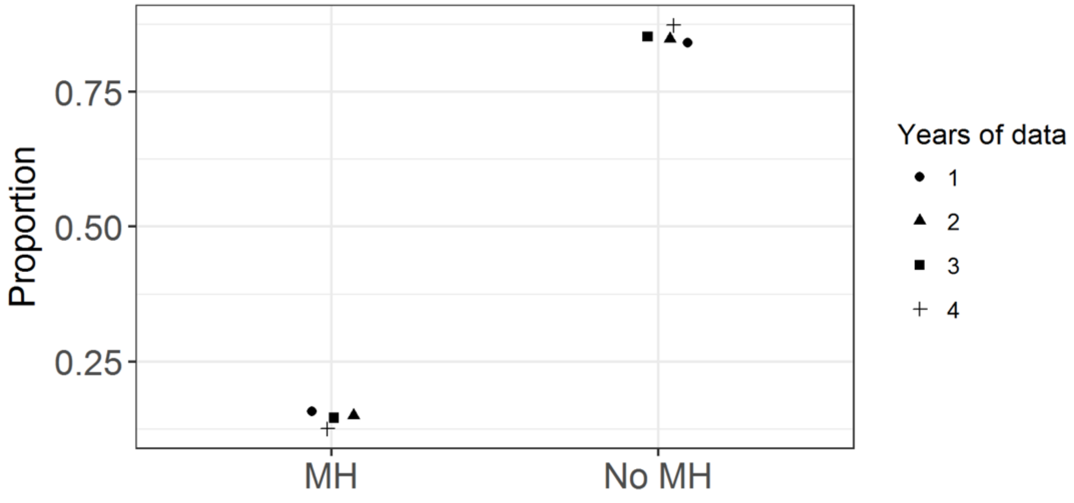

Supplementary figure 4: proportion of our cohort population in each deprivation WIMD) quintile, by the number of years contributed to the analysis. Quintile 5 is most deprived.

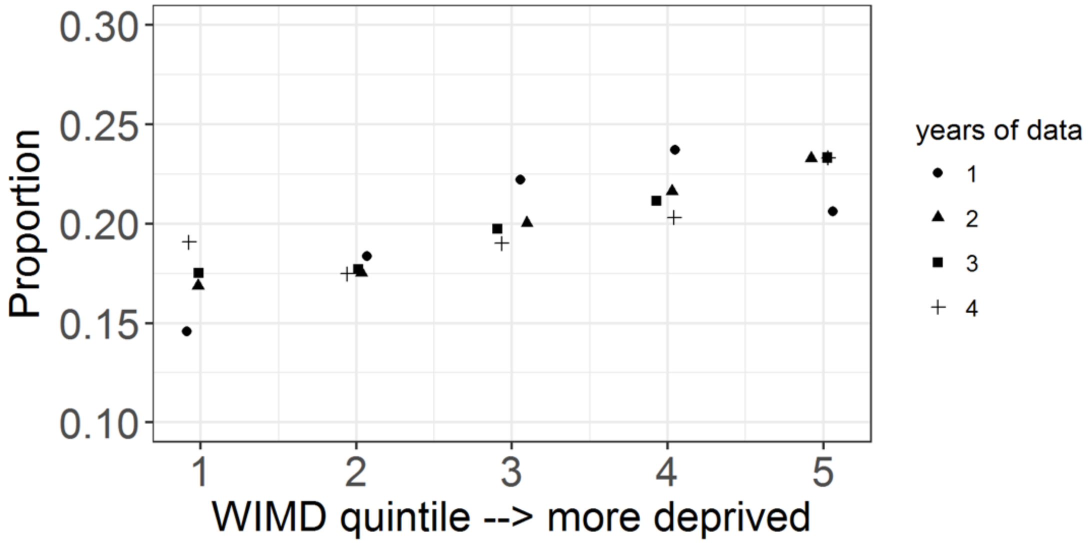

Supplementary figure 5: proportion of our cohort population by gender and the number of years contributed to the analysis.

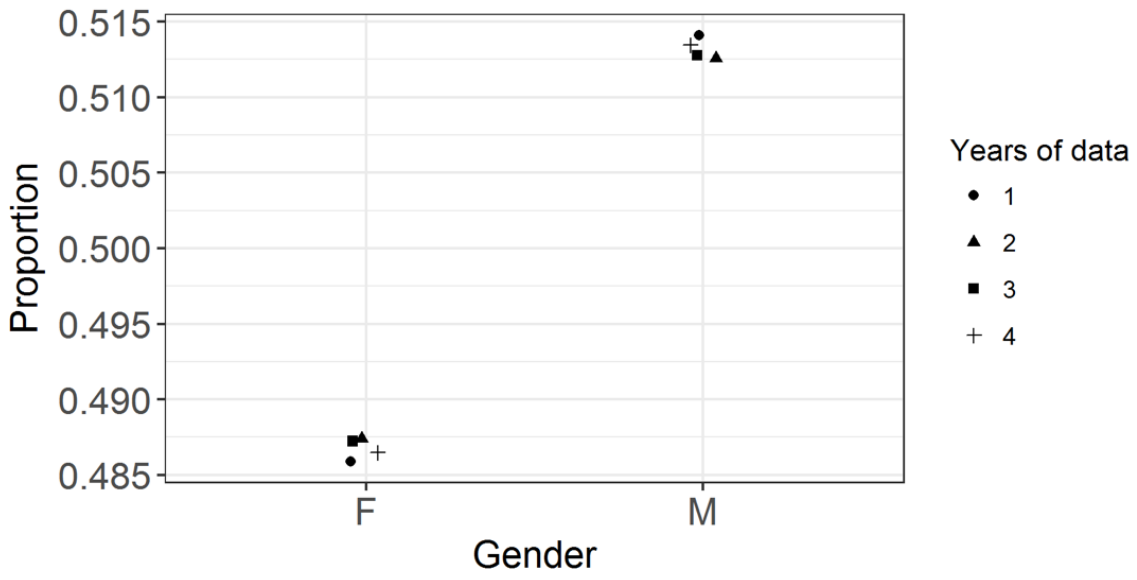

Supplementary figure 6: Boxplots describing the distribution of absence rates in those not excluded (0) and those excluded (1).

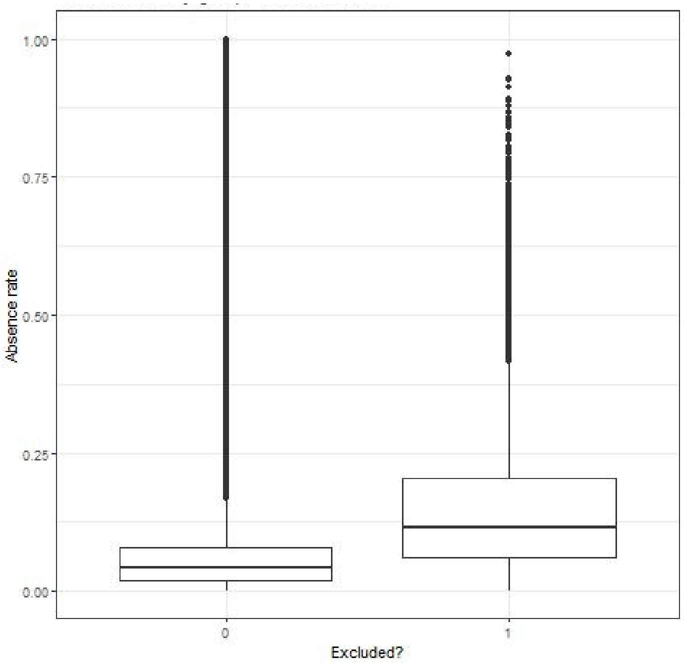

Supplementary Table 1: Datasets used in our analysis.

Database	Description	Covariates and outcomes
The Welsh Demographic Service Dataset WDSD[1]	An administrative register that includes anonymised demographic characteristics of people registered with GP practices in Wales	age, sex (male or female)
Welsh Index of Multiple Deprivation (WIMD)[2]	The official measure of deprivation for close to 2000 small areas in Wales. WIMD is made up of eight separate domains of deprivation: income; employment; health; education; housing; access to services; environment; and community safety.	Deprivation quintiles, 5 is the most deprived.
The Office for National Statistics (ONS) deaths register[3]	A register of all deaths relating to Welsh residents, including those that died outside Wales	
The Welsh Longitudinal General Practice Database – WLGP[4]	contains attendance and clinical information for all general practices who agreed to share their data (at the time of analysis 330/412 General Practices were registered with SAIL covering ~79% of the Welsh population). Clinical information including symptoms, investigations, diagnoses and prescribed medication is coded using Read Codes, a standard vocabulary for clinicians to record patient findings and procedures, in health and social care IT systems across primary and secondary care in the United Kingdom	Mental health
Patient Episode Database for Wales (PEDW)[5]	contains all inpatient and day case activity undertaken in NHS Wales plus data on Welsh residents treated in English Trusts. This includes demographic, clinical and administrative detail, such as age and sex of patient diagnoses and operative procedures. Clinical information in PEDW is coded using ICD-10 (World Health Organisation, 2016), an international standard for diagnostic classification of diseases and other health conditions	Mental health
Welsh Government Education Dataset[6]	includes records for all children registered at state schools in Wales or educated in settings other than school. It holds information on schools, examination results, attendance per year, exclusions per year, eligibility for free school meals, receipt of statement of special educational needs (SEN) and education other than at school (EOTAS). Exclusion records were available from academic year 2012/13. Exclusions were categorised within the dataset as permanent, fixed or lunchtime. A child will have Special Educational Needs (SEN) if they have a learning difficulty or disability which requires special education provision to be made for them. There are several levels of provision in schools. There are several levels of provision in schools including curriculum modification, targeted differentiation, small group provision, additional support in class and access to specialised equipment.	Absenteeism and exclusions per academic year per pupil, SEN status.

1 Public Health Wales Observatory. (2017a). *Welsh Demographics Service (WDS)*. http://www.publichealthwalesobservatory.wales.nhs.uk/wds
2 Public Health Wales Observatory. (2017b). *Welsh Index of Multiple Deprivation (WIMD).* http://www.publichealthwalesobservatory.wales.nhs.uk/wimd
3 *Annual District Death Extract (ADDE)*. (2017). https://saildatabank.com/saildata/sail-datasets/annual-district-death-extract-adde/
4 Primary Care GP dataset. (2018). https://saildatabank.com/saildata/sail-datasets/primary-care-gp-dataset/
5 PEDW. (2018). http://www.infoandstats.wales.nhs.uk/page.cfm?pid=41009&orgid=869
6 https://data.ukserp.ac.uk/Asset/View/35

Supplementary table 2: references for clinical codes.

Learning difficulties[1,1a]
ADHD[2]
ASD[3]
Anxiety[4,6]
Bipolar disorder[5]
Conduct disorder[6]
Depression[4, 4a]
Eating disorders[8,9]
Other psychotic disorders[5]<br>**Read codes**: Eu23z, Eu231, Eu230, Eu232, Eu23., Eu328, Eu23y, Eu233, Eu32A, Eu333, Eu323, Eu329, E131., E133., E11.., E13y1, 146H., E1y.., E13z., E1z.., 9H8.., E11zz, E13.., E13y., E13yz, E11z., E134., E13y0, E132., E130., E1134, 8HHs., E1124, E11z0<br>**ICD-10**: F23.., F230., F231., F232., F233., F238., F239., F28.., F28X., F29.., F29X., F30.., F300., F301., F308., F309., F323., F333., F38.., F380., F381., F388., F39.., F39X.
Schizophrenia[5]
Alcohol misuse[11]
Drug misuse[11]
Self-harm[5,9,10]

1 Burt, A., Maconochie, N., Doyle, P., & Roman, E. (2004). Learning difficulties in children born to male UK nuclear industry employees; analysis from the nuclear industry family study. Occupational and Environmental Medicine, 61(9), 786–789. https://doi.org/10.1136/oem.2004.012906

1a Brophy, Sinead et al. "Characteristics of Children Prescribed Antipsychotics: Analysis of Routinely Collected Data." Journal of child and adolescent psychopharmacology vol. 28,3 (2018): 180-191. doi:10.1089/cap.2017.0003

2 Langley, 2019, private com. (?)

3 Underwood, J. F. G., Kendall, K. M., Berrett, J., Lewis, C., Anney, R., van den Bree, M. B. M., & Hall, J. (2019). Autism spectrum disorder diagnosis in adults: phenotype and genotype findings from a clinically derived cohort. *British Journal of Psychiatry*, *215*(5), 647–653. https://doi.org/10.1192/bjp.2019.30

4 Cornish, R. P., John, A., Boyd, A., Tilling, K., & Macleod, J. (2016). Defining adolescent common mental disorders using electronic primary care data: A comparison with outcomes measured using the CIS-R. BMJ Open, 6(12). https://doi.org/10.1136/bmjopen-2016-013167;

4a John, A., Marchant, A. L., Fone, D. L., McGregor, J. I., Dennis, M. S., Tan, J. O. A., & Lloyd, K. (2016). Recent trends in primary-care antidepressant prescribing to children and young people: an e-cohort study. Psychological Medicine, 46(16), 3315–3327. https://doi.org/10.1017/S0033291716002099

5 John, Ann, McGregor, J., Jones, I., Lee, S. C., Walters, J. T. R., Owen, M. J., O'Donovan, M., DelPozo-Banos, M., Berridge, D., & Lloyd, K. (2018). Premature mortality among people with severe mental illness — New evidence from linked primary care data. Schizophrenia Research, 199, 154–162. https://doi.org/10.1016/j.schres.2018.04.009

6 John, Ann, Wood, S., Rees, S., Wang, T., & Marchant, A. (2019). Mental Healthcare in Young People and Young Adults. https://www.ncepod.org.uk/2019ypmh.html

7 Demmler, J. C., Brophy, S. T., Marchant, A., John, A., & Tan, J. O. A. (2019). Shining the light on eating disorders, incidence, prognosis and profiling of patients in primary and secondary care: national data linkage study. The British Journal of Psychiatry, 1–8. https://doi.org/10.1192/bjp.2019.153

8 Wood, S., Marchant, A., Allsopp, M., Wilkinson, K., Bethel, J., Jones, H., & John, A. (2019). Epidemiology of eating disorders in primary care in children and young people: a Clinical Practice Research Datalink study in England. BMJ Open, 9(8), e026691. https://doi.org/10.1136/bmjopen-2018-026691

9 Thomas, K. H., Davies, N., Metcalfe, C., Windmeijer, F., Martin, R. M., & Gunnell, D. (2013). Validation of suicide and self-harm records in the clinical practice research datalink. *British Journal of Clinical Pharmacology*, *76*(1), 145–157. https://doi.org/10.1111/bcp.12059

10 John, Ann. (2015). Talk to me Annexes – Suicide and Self Harm Prevention Strategy and Action Plan for Wales 2015-2020 2. https://gov.wales/sites/default/files/publications/2019-06/talk-to-me-2-annexes-suicide-and-self-harm-prevention-strategy-and-action-plan-for-wales-2015-2020_0.pdf

11 S. Rees, private communication, to be submitted, see p28 below

Supplementary table 3: Number of individuals having one or more mental disorders, their sex and statistics for the age at first diagnosis.

Mental disorder	Number of pupils with disorder[1]	Number of girls with disorder[1]	Mode of age at first diagnosis[2]	Inter-quantile 1	Inter-quantile 3	Number of people with one disorder	people with comorbidities	People with SEN (% of total)	Number diagnosed after school age (% of total)
ADHD	8,199	1,625	8	7	11	4,515	3,684	7196 (88%)	229 (3%)
ASD	7,055	1,516	9	6	12	3,786	3,269	6400 (91%)	357 (5%)
Learning difficulties	3,867	1,272	3	4	11	2,303	1,564	3433 (89%)	159 (4%)
Conduct disorder	4,417	1,434	7	5	11	2,117	2,300	3041 (69%)	46 (1%)
Depression	22,888	15,198	17	15	18	12,141	10,747	7982 (35%)	11710 (50%)
Anxiety	19,727	13,118	17	13	18	11,138	8,589	6662 (34%)	8100 (41%)
Eating disorder	1,504	1,320	15	13	16	617	887	404 (27%)	351 (23%)
Bipolar disorder	164	132	19	16	19	7	157	76 (46%)	112 (68%)
Schizophrenia	217	80	18	16	19	15	202	109 (50%)	149 (69%)
other psychotic disorders	327	159	18	15	18	37	290	164 (50%)	189 (58%)
Drug misuse	1,990	774	17	15	18	454	1,536	1024 (51%)	1069 (54%)
Alcohol misuse	2,434	1,313	15	14	17	1,083	1,351	1016 (42%)	845 (35%)
Self-harm	8,706	6,652	15	14	16	2,805	5,901	3631 (42%)	2160 (35%)

1 Pupils may have more than one condition

2 highest number of people with the condition are first diagnosed at this age.

Supplementary table 4: Odds ratios for absenteeism by sex, age or deprivation in stratified populations that included individuals with each of the mental disorders listed. P<0.0001 unless otherwise specified.

MH condition	OR (95%CI) (males) p	OR (95%CI) age p	OR (95%CI) deprivation (2nd vs 1st quintile), p	OR (95%CI) deprivation (5th vs 1st quintile), p
ADHD *(N=8199)*	**0.8 (0.8, 0.9) 4e-04**	**1.09 (1.08, 1.1)**	**1.3 (1.1, 1.6) 6e-04**	**2.2 (1.9, 2.5)**
ASD *(N*= 7055*)*	**0.8 (0.7, 0.9)**	**1.01 (1, 1.03) 0.0432**	**1.3 (1.1, 1.5) 0.0021**	**2.0 (1.7, 2.3)**
Learning difficulties (N= 3867)	**0.8 (0.7, 0.9)**	1 (0.98, 1.01) 0.7806	**1.5 (1.2, 1.8) 5e-04**	**2.2 (1.8, 2.7)**
Conduct disorder (N= 4417)	**0.8 (0.7, 0.9) 5e-04**	**1.07 (1.06, 1.09)**	1.2 (1, 1.5) 0.053	**1.5 (1.3, 1.9)**
Depression (*N=22,888*)	**0.8 (0.8, 0.9)**	**1.1 (1.09, 1.11)**	**1.3 (1.2, 1.4)**	**2.3 (2.2, 2.5)**
Anxiety (N= 19,727)	1.0 (0.9, 1.0) 0.072	**1.07 (1.06, 1.08)**	**1.5 (1.4, 1.6)**	**2.7 (2.5, 2.9)**
Eating disorders (*N=1,504*)	1.0 (0.8, 1.4) 0.79	**1.13 (1.09, 1.18)**	**1.6 (1.2, 2.2) 0.0014**	**2.6 (2.0, 3.5)**
Bipolar disorder (N=164)	0.7 (0.4, 1.4) 0.32	0.99 (0.85, 1.15) 0.9069	NA	NA
Schizophrenia (N=217)	0.8 (0.5, 1.3) 0.38	1.11 (0.98, 1.25) 0.0917	NA	NA
Other psychotic disorders (N=327)	**0.7 (0.5, 1.0) 0.036**	1.05 (0.96, 1.14) 0.2592	0.6 (0.3, 1.2) 0.13	1.4 (0.8, 2.7) 0.26
Drug misuse (N= 1,990)	**0.6 (0.6, 0.7)**	**1.15 (1.1, 1.2)**	1.3 (1.0, 1.7) 0.096	**2.1 (1.6, 2.7)**
Alcohol misuse (N= 2,434)	**0.7 (0.6, 0.8)**	**1.13 (1.09, 1.17)**	**1.5 (1.1, 1.9) 0.0082**	**2.8 (2.2, 3.6)**
Self-harm (N= 8,706)	1.0 (0.9, 1.0) 0.29	**1.16 (1.15, 1.18)**	**1.3 (1.1, 1.5) 1e-04**	**2.0 (1.8, 2.3)**

Supplementary Table 5: distribution of pupils with comorbidities.

Number of morbidities	Number of pupils
1	41,018
2	12,096
3	3,495
4	957
5	247
6	87
>=7	30

Supplementary table 6: Odds ratios for absenteeism in a model adjusted for sex, age deprivation and the number of comorbidities as a variable.

MH condition	OR (95%CI) adjusted for sex, age, deprivation and comorbidities	OR (95%CI) for the comorbidity covariate
ADHD	1.3 (1.2, 1.4)	1.3 (1.2, 1.3)
ASD	1.2 (1.1, 1.3)	1.3 (1.3, 1.4)
Learning difficulties	1.4 (1.3, 1.6)	1.2 (1.2, 1.3)
Conduct disorder	1.7 (1.6, 1.9)	1.2 (1.2, 1.3)
Depression	1.8 (1.8, 1.9)	1.3 (1.3, 1.3)
Anxiety	1.4 (1.3, 1.5)	1.4 (1.4, 1.5)
Eating disorder	1.1 (0.9, 1.3) p=0.3	1.4 (1.3, 1.5)
Bipolar disorder	5.4 (2.7, 10.6)	1.0 (0.9, 1.2)
Schizophrenia	2.6 (1.5, 4.7) p=0.001	1.2 (1.0, 1.3)
Other psychiatric disorders	1.8 (1.1, 2.8) p=0.02	1.2 (1.1, 1.3)
Drug misuse	2.8 (2.4, 3.3)	1.2 (1.1, 1.2)
Alcohol misuse	1.8 (1.6, 2.1)	1.3 (1.2, 1.4)
Self-harm	2.3 (2.1, 2.4)	1.2 (1.2, 1.3)

Supplementary table 7: Odds ratios for absenteeism in a model adjusted for sex, age deprivation with interaction between SEN status and mental disorder. P<0.0001 unless otherwise specified.

MH condition	adjusted for sex, age, deprivation and interaction with SEN	OR (95%CI) for SEN covariate	OR (95%CI) SEN x EVENT
ADHD *(N=8199)*	2.2 (2.0, 2.4)	1.6 (1.6, 1.6)	0.65 (0.59, 0.71)
ASD *(N=* 7055*)*	2.4 (2.2, 2.6)	1.6 (1.6, 1.6)	0.59 (0.54, 0.66) p=0.008
Learning difficulties (N= 3867)	1.8 (1.6, 2.1)	1.6 (1.6, 1.6)	0.82 (0.71, 0.95)
Conduct disorder (N= 4417)	2.9 (2.7, 3.1)	1.6 (1.6, 1.6)	0.67 (0.61, 0.73)
Depression (*N=22,888*)	2.8 (2.8, 2.9)	1.6 (1.6, 1.6)	0.89 (0.85, 0.94)
Anxiety (N= 19,727)	2.4 (2.3, 2.5)	1.6 (1.6, 1.6)	1.0 (0.95, 1.05) p=0.8
Eating disorders (*N=1,504*)	2.1 (1.9, 2.3)	1.6 (1.6, 1.6)	1.07 (0.89, 1.28) p=0.5
Bipolar disorder (N=164)	6.1 (4.4, 8.6)	1.6 (1.6, 1.6)	0.61 (0.38, 0.98) p=0.04
Schizophrenia (N=217)	4.6 (3.4, 6.1)	1.6 (1.6, 1.6)	0.68 (0.44, 1.04) p=0.07
Other psychotic disorders (N=327)	3.4 (2.7, 4.3)	1.6 (1.6, 1.6)	0.7 (0.51, 0.97)
Drug misuse (N= 1,990)	4.2 (3.8, 4.7)	1.6 (1.6, 1.6)	0.78 (0.68, 0.9)
Alcohol misuse (N= 2,434)	3.2 (2.9, 3.5)	1.6 (1.6, 1.6)	0.92 (0.81, 1.05) p=0.2
Self-harm (*N= 8,706*)	3.5 (3.3, 3.6)	1.6 (1.6, 1.6)	0.82 (0.76, 0.87)

Supplementary table 8: Odds ratios for exclusion by sex, age or deprivation in stratified populations that included individuals with each of the mental disorders listed. P<0.0001 unless otherwise specified. NA means not enough individuals. In the population with no disorder OR for males was 3.3, OR for age was 1.3, for quintile 2 OR=1.6, for quintile 5 OR=4.7

MH condition	OR (95%CI) (males) p	OR (95%CI) age p	OR (95%CI) deprivation (2nd vs 1st quintile), p	OR (95%CI) deprivation (5th vs 1st quintile), p
ADHD *(N=8199)*	1.5 (1.3, 1.8)	1.12 (1.1, 1.14)	1.2 (0.9, 1.5) 0.2	1.8 (1.5, 2.2)
ASD *(N=* 7055*)*	1.8 (1.4, 2.3)	1.09 (1.06, 1.12)	1.2 (0.8, 1.7) 0.3	1.9 (1.4, 2.5)
Learning difficulties (N= 3867)	2.1 (1.5, 3)	1.15 (1.1, 1.2)	1.1 (0.7, 2) 0.6	1.8 (1.1, 3.0) 0.02
Conduct disorder (N= 4417)	1.7 (1.4, 2.1)	1.13 (1.1, 1.15)	1.0 (0.7, 1.4) 0.9	1.4 (1.1, 1.9) 0.02
Depression (*N=22,888*)	1.9 (1.7, 2.1)	1.12 (1.1, 1.14)	1.2 (1, 1.4) 0.09	2.3 (2.0, 2.7)
Anxiety (N= 19,727)	2.0 (1.8, 2.2)	1.17 (1.15, 1.2)	1.3 (1, 1.7) 0.03	3.3 (2.7, 4.0)
Eating disorders (*N=1,504*)	2.3 (1.3, 4.2) 0.006	1.19 (1.08, 1.3) 3e-04	0.3 (0.1, 1.1) 0.08	2.8 (1.3, 6.0) 0.008
Bipolar disorder (N=164)	2.0 (0.9, 4.7) 0.09	0.87 (0.73, 1.05) 0.1	NA	NA
Schizophrenia (N=217)	2.2 (1.1, 4.6) 0.04	0.96 (0.84, 1.09) 0.5	NA	NA
Other psychotic disorders (N=327)	2.9 (1.5, 5.9) 0.003	1.1 (0.99, 1.22) 0.08	1.6 (0.5, 4.7) 0.4	0.7 (0.2, 2.0) 0.5
Drugs misuse (N= 1,990)	1.4 (1.2, 1.7)	1.03 (0.99, 1.08) 0.1	1.0 (0.7, 1.3) 0.8	1.5 (1.1, 2.0) 0.006
Alcohol misuse (N= 2,434)	1.4 (1.1, 1.6) 0.002	1.01 (0.97, 1.04) 0.8	1.2 (0.8, 1.9) 0.4	2.5 (1.7, 3.6)
Self-harm (*N= 8,706*)	2.5 (2.2, 2.8)	1.12 (1.09, 1.14)	1.1 (0.8, 1.3) 0.7	1.7 (1.4, 2.1)

Supplementary table 9: Odds ratios for exclusion in a model adjusted for sex, age deprivation and the number of comorbidities. P<0.0001 unless otherwise specified.

**MH condition**	**OR (95%CI) adjusted for sex, age, deprivation and comorbidities**	**OR (95%CI) for the comorbidity covariate**
ADHD *(N=8199)*	4.2 (3.7, 4.6)	1.2 (1.2, 1.3)
ASD *(N=* 7055*)*	1.3 (1.1, 1.5) p=0.004	1.4 (1.3, 1.5)
Learning difficulties (N= 3867)	0.6 (0.5, 0.9) p=0.002	1.7 (1.5, 1.8)
Conduct disorder (N= 4417)	3.3 (2.9, 3.9)	1.3 (1.3, 1.4)
Depression (*N=22,888*)	1.4 (1.3, 1.5)	1.6 (1.5, 1.6)
Anxiety (N= 19,727)	0.8 (0.7, 0.9)	1.8 (1.7, 1.9)
Eating disorders (*N=1,504*)	1.0 (0.6, 1.6) p=0.9	1.3 (1.1, 1.5)
Bipolar disorder (N=164)	2.9 (1.0, 8.5) p=0.06	1.3 (1.0, 1.6)
Schizophrenia (N=217)	2.7 (1.0, 6.8) p=0.04	1.3 (1.0, 1.6)
Other psychotic disorders (N=327)	1.9 (0.8, 4.3) p=0.1	1.3 (1.0, 1.6)
Drug misuse (N= 1,990)	7.5 (6.2, 9.1)	1.2 (1.1, 1.2)
Alcohol misuse (N= 2,434)	2.8 (2.3, 3.4)	1.4 (1.3, 1.5)
Self-harm (*N= 8,706*)	3.6 (3.2, 4.1)	1.3 (1.2, 1.4)

Table 10: Odds ratios of exclusions for a model with interaction between SEN status and disorder. p<0.0001 unless otherwise specified.

MH condition	OR (95%CI) adjusted for sex, age, deprivation and interaction with SEN	OR (95%CI) for SEN covariate	OR (95%CI) SEN x EVENT
ADHD *(N=8199)*	7.1 (6.2, 8.2)	2.9 (2.8, 3)	0.48 (0.41, 0.56)
ASD (*N*= 7055*)*	2.9 (2.2, 3.7)	2.9 (2.8, 3)	0.51 (0.38, 0.67)
Learning difficulties (N= 3867)	1.4 (0.9, 2.3) p=0.1	2.9 (2.8, 3)	0.69 (0.43, 1.11) p=0.1
Conduct disorder (N= 4417)	5.4 (4.7, 6.3)	2.9 (2.8, 3)	0.73 (0.61, 0.88)
Depression (*N=22,888*)	3.2 (2.9, 3.4)	2.9 (2.8, 3.1)	0.83 (0.76, 0.92) p=0.0002
Anxiety (N= 19,727)	2.3 (2.1, 2.5)	2.9 (2.8, 3)	0.9 (0.8, 1.02) p=0.09
Eating disorders (*N=1,504*)	1.8 (1.3, 2.5) p=0.0006	2.9 (2.8, 3)	1.0 (0.61, 1.63) p=1
Bipolar disorder (N=164)	10.6 (5.9, 19)	2.9 (2.8, 3)	0.32 (0.15, 0.71) p=0.005
Schizophrenia (N=217)	7.5 (4.4, 12.6)	2.9 (2.8, 3)	0.55 (0.29, 1.03) p=0.06
Other psychotic disorders (N=327)	5.6 (3.5, 9)	2.9 (2.8, 3)	0.41 (0.21, 0.8) 0.009
Drug misuse (N= 1,990)	12.2 (10.7, 14.0)	2.9 (2.8, 3)	0.6 (0.5, 0.71)
Alcohol misuse (N= 2,434)	6.1 (5.2, 7.1)	2.9 (2.8, 3)	0.83 (0.67, 1.02) p=0.07
Self-harm (*N= 8,706*)	6.9 (6.3, 7.5)	2.9 (2.8, 3)	0.69 (0.62, 0.78)

Supplementary table 11: Numbers of pupils and of absentees per year group and disorder. These counts were used to create figure 2.

Disorder	Age (years, as proxy for school year groups)	Absentees (N)	Total (N)
ADHD	7	415	2,035
	8	435	2,317
	9	514	2,437
	10	496	2,468
	11	547	2,517
	12	600	2,480
	13	747	2,457
	14	801	2,410
	15	823	2,463
	16	806	2,299
Alcohol misuse	8	6	22
	9	18	72
	10	40	165
	11	85	317
	12	152	513
	13	245	740
	14	399	1,013
	15	583	1,389
	16	717	1,725
Anxiety	7	166	801
	8	341	1,448
	9	591	2,262
	10	776	3,184
	11	1,016	4,169
	12	1,369	5,359

	13	2,029	6,735
	14	2,770	8,311
	15	3,421	10,358
	16	4,085	12,133
ASD	7	483	2,016
	8	458	2,079
	9	517	2,178
	10	500	2,167
	11	552	2,236
	12	507	2,118
	13	565	2,056
	14	553	1,957
	15	513	1,940
	16	480	1,766
Bipolar disorder	13	23	41
	14	31	59
	15	49	90
	16	66	131
Conduct disorder	7	244	957
	8	285	1,155
	9	333	1,241
	10	333	1,273
	11	346	1,332
	12	408	1,340
	13	546	1,404
	14	590	1,402
	15	618	1,472
	16	533	1,416
Depression	7	63	232
	8	139	544

	9	284	1,126
	10	508	2,105
	11	811	3,291
	12	1,240	4,968
	13	2,345	7,148
	14	3,627	9,755
	15	4,989	13,299
	16	6,327	16,351
Drug misuse	9	9	37
	10	27	90
	11	55	183
	12	114	338
	13	247	581
	14	379	838
	15	554	1,195
	16	680	1,404
Eating disorder	7	6	31
	8	15	74
	9	27	138
	10	37	229
	11	61	327
	12	69	462
	13	141	591
	14	209	694
	15	253	844
	16	288	906
Learning difficulties	7	258	1,011
	8	266	1,080
	9	300	1,132
	10	255	1,115

	11	285	1,125
	12	225	1,045
	13	284	1,111
	14	315	1,086
	15	296	1,155
	16	325	1,168
No disorder	7	15,493	120,672
	8	14,584	119,454
	9	14,316	115,874
	10	13,688	110,894
	11	12,708	105,854
	12	12,064	101,752
	13	15,589	98,944
	14	16,879	96,151
	15	16,772	95,929
	16	17,389	93,907
Other psychotic disorders	10	8	23
	11	11	40
	12	15	66
	13	40	106
	14	55	138
	15	67	180
	16	94	227
Schizophrenia	11	7	21
	12	15	37
	13	25	56
	14	42	86
	15	53	119
	16	74	159
Self-harm	7	34	84

	8	75	264
	9	158	585
	10	289	1,130
	11	457	1,805
	12	687	2,612
	13	1,242	3,417
	14	1,682	4,110
	15	2,227	4,949
	16	2,380	5,158

Supplementary table 12: Numbers of pupils and of excluded pupils per year group and disorder. These counts were used to create figure 3.

Disorder	Age (years, as proxy for school year groups)	Excluded (N)	Total (N)
ADHD	7	72	2,038
	8	124	2,343
	9	130	2,474
	10	135	2,532
	11	138	2,570
	12	292	2,589
	13	356	2,555
	14	374	2,483
	15	357	2,529
	16	226	2,376
Alcohol misuse	11	9	320
	12	45	517
	13	89	747
	14	150	1,026
	15	197	1,404
	16	145	1,741
Anxiety	8	8	1,449
	9	22	2,265
	10	24	3,190
	11	28	4,176
	12	90	5,385
	13	226	6,775
	14	356	8,341
	15	567	10,388
	16	481	12,195
ASD	7	38	2,018

	8	64	2,123
	9	63	2,231
	10	47	2,232
	11	45	2,288
	12	102	2,270
	13	120	2,177
	14	126	2,045
	15	108	1,986
	16	86	1,826
Bipolar disorder	13	7	41
	14	9	59
	15	16	92
	16	7	133
Conduct disorder	7	28	958
	8	51	1,160
	9	57	1,244
	10	67	1,298
	11	55	1,348
	12	131	1,372
	13	185	1,437
	14	207	1,433
	15	208	1,490
	16	142	1,435
Depression	9	8	1,125
	10	18	2,107
	11	31	3,296
	12	128	4,983
	13	351	7,173
	14	602	9,779
	15	984	13,328

	16	835	16,437
Drug misuse	10	6	90
	11	8	183
	12	46	341
	13	123	586
	14	205	848
	15	297	1,214
	16	255	1,423
Eating disorder	13	18	593
	14	15	694
	15	20	842
	16	21	916
Learning difficulties	7	9	1,014
	8	13	1,107
	9	13	1,155
	10	20	1,154
	11	17	1,164
	12	31	1,141
	13	45	1,189
	14	48	1,139
	15	55	1,181
	16	40	1,207
No disorder	7	141	120,673
	8	202	119,511
	9	286	115,670
	10	363	110,964
	11	408	105,929
	12	1,167	101,959
	13	1,815	99,116
	14	2,284	96,262

	15	2,770	95,984
	16	2,064	94,173
Other psychotic disorders	13	9	107
	14	17	138
	15	21	182
	16	17	230
Schizophrenia	13	7	56
	14	16	87
	15	19	119
	16	21	160
Self-harm	9	12	586
	10	12	1,133
	11	36	1,807
	12	139	2,623
	13	311	3,439
	14	443	4,135
	15	605	4,985
	16	379	5,207

Supplementary table 13: Sensitivity analysis of absenteeism by neurodevelopmental disorder, mental disorder, drugs or alcohol misuse, and self-harm in the cohort that includes people with no linked healthcare data (N= 437,412). All results are highly significant (p<0·0001).

Mental disorder	OR (95% CI)	aOR (95% CI) *
ADHD (N=8199)	2.2 (2.1, 2.3)	2.0 (1.9, 2.1)
ASD (N= 7055)	2.1 (2.0, 2.2)	2.0 (1.9, 2.1)
Learning difficulties (N= 3867)	2.1 (2.0, 2.3)	2.0 (1.9, 2.1)
Conduct disorder (N= 4417)	3.0 (2.9, 3.2)	2.6 (2.5, 2.8)
Depression (*N=22,888*)	3.4 (3.3, 3.5)	2.9 (2.8, 2.9)
Anxiety (N= 19,727)	2.8 (2.7, 2.8)	2.5 (2.4, 2.6)
Eating disorders (*N=1,504*)	2.2 (2.0, 2.5)	2.2 (2.0, 2.4)
Bipolar disorder (N=164)	6.5 (5.0, 8.5)	5.5 (4.2, 7.3)
Schizophrenia (N=217)	5.0 (4.0, 6.2)	4.3 (3.4, 5.3)
Other psychotic disorders (N=327)	3.7 (3.0, 4.4)	3.2 (2.6, 3.9)
Drug misuse (N= 1,990)	5.0 (4.7, 5.4)	4.2 (3.9, 4.5)
Alcohol misuse (N= 2,434)	3.9 (3.6, 4.2)	3.3 (3.1, 3.5)
Self-harm (*N= 8,706*)	4.0 (3.9, 4.2)	3.5 (3.3, 3.6)

*Adjusted for sex, age, and deprivation

Supplementary table 14: Sensitivity analysis of exclusions by neurodevelopmental disorder, mental disorder, drugs or alcohol misuse, and self-harm in the cohort that includes people with no linked healthcare data (N= 437,412). All results are highly significant (p<0·0001). *Adjusted for sex, age, and deprivation

Mental disorder	OR (95% CI)	aOR (95% CI) *
ADHD (N=8199)	9.0 (8.6, 9.6)	6.1 (5.7, 6.4)
ASD (N= 7055)	3.6 (3.3, 4.0)	2.6 (2.4, 2.9)
Learning difficulties (N= 3867)	2.4 (2.1, 2.8)	1.8 (1.5, 2.0)<br>P= 0.74
Conduct disorder (N= 4417)	8.7 (8.0, 9.4)	6.0 (5.5, 6.5)
Depression (*N=22,888*)	5.0 (4.8, 5.3)	3.2 (3.1, 3.4)
Anxiety (N= 19,727)	3.3 (3.1, 3.5)	2.5 (2.3, 2.6)
Eating disorders (*N=1,504*)	1.8 (1.4, 2.3)	1.9 (1.5, 2.5)
Bipolar disorder (N=164)	10.6 (7.3, 15.4)	7.4 (4.9, 11.0)
Schizophrenia (N=217)	14.4 (10.5, 19.8)	6.6 (4.7, 9.2)
Other psychotic disorders (N=327)	7.7 (5.6, 10.7)	4.2 (3.0, 5.9)
Drug misuse (N= 1,990)	22.9 (21.1, 24.9)	11.0 (10.0, 12.1)
Alcohol misuse (N= 2,434)	11.2 (10.2, 12.4)	6.3 (5.7, 7.1)
Self-harm (*N= 8,706*)	8.3 (7.8, 8.8)	6.8 (6.3, 7.2)

Supplementary table 15: Goodness-of-fit parameter (QIC) for models when sequentially adding covariates.

model	QIC (exclusions)	QIC (absenteeism)
crude	1153277.45	229837.28
Adjusted for sex	1153250.90	225699.66
Adjusted for sex, Age	1148765.23	217717.50
Adjusted for sex, Age, deprivation	1122861.9	213823.2

Substance misuse - Read v2 codes

We compiled a list of Read v2 codes with advice from a specialist substance misuse nurse based in a GP practice and with expertise in using Read v2 codes to record SUD. We browsed Read code reference data to identify any missing codes. The code list included codes for diagnoses, symptoms/observations, medications, behaviors (e.g., 'injecting drug user'), referrals and contacts with other services. We included codes that on their own designated CC. We included codes designating misuse of alcohol or illegal drugs but not prescribed medication or tobacco. We also included codes designating a MHD due to substance use, which we classified as designating a CC without the requirement for an associated MHD code.

We included only those prescriptions which were relevant to substance use or addiction treatment: prescription of disulfiram, naltrexone, lofexidine, acamprosate and methadone without associated value of units. The final Read v2 SUD code list (including codes designating a history of a condition) contained 876 codes:

- 167 alcohol codes
- 566 drug codes
- 143 CC codes
- Of the above codes, 80 refer to a history of a SUD

Substance use disorders ICD10 codes:

ICD10 codes were initially identified by cross-mapping the Read code list with ICD10 reference data. The ICD10 SUD code list contained 221 codes:

- 68 alcohol codes
- 54 drug codes
- 99 CC codes

Deprivation scores

Assigning a yearly deprivation score: our dataset includes information on the deprivation level and time periods at that level for each individual. When deprivation data were available for a given academic year we used it, and if a pupil moved between quintiles, we used a time weighted mean. Where deprivation scores were available for a pupil but not for some academic years, we imputed a weighted mean from available years. If deprivation scores were not available for any of the academic years but was available before or after our time-period of focus, we imputed a weighted mean of available deprivation scores into all available academic years.

The RECORD statement – checklist of items, extended from the STROBE statement, that should be reported in observational studies using routinely collected health data.

	Item No.	STROBE items	Location in manuscript where items are reported	RECORD items	Location in manuscript where items are reported
**Title and abstract**					
	1	(a) Indicate the study's design with a commonly used term in the title or the abstract (b) Provide in the abstract an informative and balanced summary of what was done and what was found		RECORD 1.1: The type of data used should be specified in the title or abstract. When possible, the name of the databases used should be included.<br><br>RECORD 1.2: If applicable, the geographic region and timeframe within which the study took place should be reported in the title or abstract.<br><br>RECORD 1.3: If linkage between databases was conducted for the study, this should be clearly stated in the title or abstract.	Title and abstract (p2)
**Introduction**					
Background rationale	2	Explain the scientific background and rationale for the investigation being reported			Abstract and introduction(p2, p3)

Objectives	3	State specific objectives, including any prespecified hypotheses			Introduction (p2)
**Methods**					
Study Design	4	Present key elements of study design early in the paper			Methods, design p3
Setting	5	Describe the setting, locations, and relevant dates, including periods of recruitment, exposure, follow-up, and data collection			Study population, Data sources sections (p2,3)
Participants	6	*(a) Cohort study* - Give the eligibility criteria, and the sources and methods of selection of participants. Describe methods of follow-up<br>*Case-control study* - Give the eligibility criteria, and the sources and methods of case ascertainment and control selection. Give the rationale for the choice of cases and controls<br>*Cross-sectional study* - Give the eligibility criteria, and the sources and methods of selection of participants		RECORD 6.1: The methods of study population selection (such as codes or algorithms used to identify subjects) should be listed in detail. If this is not possible, an explanation should be provided.<br><br>RECORD 6.2: Any validation studies of the codes or algorithms used to select the population should be referenced. If validation was conducted for this study and not published elsewhere, detailed methods and results should be provided.<br><br>RECORD 6.3: If the study involved linkage of databases, consider use	Measures section<br><br>Figure 1 is a flow diagram for the cohort

		(b) Cohort study - For matched studies, give matching criteria and number of exposed and unexposed<br>*Case-control study* - For matched studies, give matching criteria and the number of controls per case		of a flow diagram or other graphical display to demonstrate the data linkage process, including the number of individuals with linked data at each stage.	
Variables	7	Clearly define all outcomes, exposures, predictors, potential confounders, and effect modifiers. Give diagnostic criteria, if applicable.		RECORD 7.1: A complete list of codes and algorithms used to classify exposures, outcomes, confounders, and effect modifiers should be provided. If these cannot be reported, an explanation should be provided.	Appendix p7
Data sources/ measurement	8	For each variable of interest, give sources of data and details of methods of assessment (measurement). Describe comparability of assessment methods if there is more than one group			Appendix p6
Bias	9	Describe any efforts to address potential sources of bias			Discussion
Study size	10	Explain how the study size was arrived at			Measures and Results/Cohort development/study cohort

Quantitative variables	11	Explain how quantitative variables were handled in the analyses. If applicable, describe which groupings were chosen, and why			Measures/covariates, outcome, explanatory variables
Statistical methods	12	(a) Describe all statistical methods, including those used to control for confounding<br>(b) Describe any methods used to examine subgroups and interactions<br>(c) Explain how missing data were addressed<br>(d) *Cohort study* - If applicable, explain how loss to follow-up was addressed<br>*Case-control study* - If applicable, explain how matching of cases and controls was addressed<br>*Cross-sectional study* - If applicable, describe analytical methods taking account of sampling strategy<br>(e) Describe any sensitivity analyses			(a) Methods/statistical analysis section p5<br>(b) Methods/statistical analysis section p5<br>(c)Results/ cohort development p6<br>(e) Methods/statistical analysis section p5
Data access and cleaning methods		..		RECORD 12.1: Authors should describe the extent to which the investigators had access to the	Methods/data sources p3

				database population used to create the study population.<br><br>RECORD 12.2: Authors should provide information on the data cleaning methods used in the study.	
Linkage		..		RECORD 12.3: State whether the study included person-level, institutional-level, or other data linkage across two or more databases. The methods of linkage and methods of linkage quality evaluation should be provided.	Methods/data sources p3
**Results**					
Participants	13	(a) Report the numbers of individuals at each stage of the study (*e.g.*, numbers potentially eligible, examined for eligibility, confirmed eligible, included in the study, completing follow-up, and analysed)<br>(b) Give reasons for non-participation at each stage.<br>(c) Consider use of a flow diagram		RECORD 13.1: Describe in detail the selection of the persons included in the study (*i.e.,* study population selection) including filtering based on data quality, data availability and linkage. The selection of included persons can be described in the text and/or by means of the study flow diagram.	Cohort development/study cohort
Descriptive data	14	(a) Give characteristics of study participants (*e.g.*, demographic, clinical, social) and information on			Cohort development/study cohort

		exposures and potential confounders<br>(b) Indicate the number of participants with missing data for each variable of interest<br>(c) *Cohort study* - summarise follow-up time (*e.g.*, average and total amount)			
Outcome data	15	*Cohort study* - Report numbers of outcome events or summary measures over time<br>*Case-control study* - Report numbers in each exposure category, or summary measures of exposure<br>*Cross-sectional study* - Report numbers of outcome events or summary measures			Table 1, table 2.
Main results	16	(a) Give unadjusted estimates and, if applicable, confounder-adjusted estimates and their precision (e.g., 95% confidence interval). Make clear which confounders were adjusted for and why they were included			Results, supplementary

		(b) Report category boundaries when continuous variables were categorized<br>(c) If relevant, consider translating estimates of relative risk into absolute risk for a meaningful time period			
Other analyses	17	Report other analyses done—e.g., analyses of subgroups and interactions, and sensitivity analyses			results
**Discussion**					
Key results	18	Summarise key results with reference to study objectives			First paragraph
Limitations	19	Discuss limitations of the study, taking into account sources of potential bias or imprecision. Discuss both direction and magnitude of any potential bias		RECORD 19.1: Discuss the implications of using data that were not created or collected to answer the specific research question(s). Include discussion of misclassification bias, unmeasured confounding, missing data, and changing eligibility over time, as they pertain to the study being reported.	Strengths and limitations
Interpretation	20	Give a cautious overall interpretation of results considering objectives, limitations, multiplicity of analyses, results from similar			Discussion, conclusion

		studies, and other relevant evidence			
Generalisability	21	Discuss the generalisability (external validity) of the study results			Implications for policy, practice and research
**Other Information**					
Funding	22	Give the source of funding and the role of the funders for the present study and, if applicable, for the original study on which the present article is based			Funding and acknowledgeme nt
Accessibility of protocol, raw data, and programming code		..		RECORD 22.1: Authors should provide information on how to access any supplemental information such as the study protocol, raw data, or programming code.	Data sharing statement

*Reference: Benchimol EI, Smeeth L, Guttmann A, Harron K, Moher D, Petersen I, Sørensen HT, von Elm E, Langan SM, the RECORD Working Committee. The REporting of studies Conducted using Observational Routinely-collected health Data (RECORD) Statement. *PLoS Medicine* 2015; in press.

*Checklist is protected under Creative Commons Attribution (CC BY) license.
